# Comparative analysis of lipid profiles and aroma characteristics in pecan oils: Probing conventional and non-conventional extraction techniques

**DOI:** 10.1016/j.fochx.2025.102470

**Published:** 2025-04-16

**Authors:** Jingtao Cui, Li Cui, Tao Zhang, Xinchen Jiang, Yaming Qian, Wuyang Huang, Haijun Zhu

**Affiliations:** a Institute of Pomology*,* Jiangsu Academy of Agricultural Sciences/Jiangsu Key Laboratory for Horticultural Crop Genetic Improvement*,* Nanjing *210014,* China; b Institute of Agro-Product Processing, Jiangsu Academy of Agricultural Sciences*,* Nanjing *210014,* China; c School of Food Science and Engineering*,* Hainan University*,* Haikou *570228,* China

**Keywords:** Pecan oil, Extraction techniques, Foodomics analysis, Lipid profile, Volatile compound

## Abstract

This study aimed to evaluate the impact of conventional (pressing extraction [PSE], solvent extraction [STE]), and non-conventional (microwave-assisted solvent extraction [MSE], subcritical fluid extraction [SBFE], supercritical fluid extraction [SPFE]) techniques on the quality of pecan oil using foodomics. The results indicated that MSE, STE, and SBFE yielded higher oil quantities. SPFE selectively extracted free fatty acids, leading to a higher acid value. MSE-derived oil exhibited the highest oxidative stability index. A total of 844 lipid molecules were identified, with glycerolipids and glycerophospholipids as the predominant classes. MSE and SBFE showed superior efficiency over conventional methods for the extraction of glycerophospholipids and saccharolipids. Additionally, 48 volatile compounds were identified, predominantly aldehydes and esters, with seven recognized as key aroma contributors. Dimethyl trisulfide had the most significant impact on the overall aroma. These findings offer a theoretical foundation for the efficient and sustainable production of pecan oil.

## Highlights

• Six methods were employed to extract pecan oil.
• MSE and SBFE achieved oil yields comparable to STE.
• MSE exhibited superior efficiency in extracting polar lipids.
• 844 lipids were detected, 94 of which were identified as differential lipids.
• Aldehydes and esters were the predominant aroma compounds in pecan oil.

## 1 Introduction

Pecan (*Carya illinoinensis* (Wangenh.) K. Koch), a member of the family Juglandaceae, is one of the oldest nuts in the world (Atanasov et al., 2018). This plant, native to the United States, was introduced to China in the early 20th century, particularly in Jiangsu, Yunnan, Zhejiang, and Anhui provinces (Zhang et al., 2015). Since 2008, the cultivation area and consumption of pecans as an oilseed crop has expanded rapidly in China. This growth is driven by the unique flavor and rich nutritional value of pecans, which are highly prized by consumers, as well as by rising walnut prices (Zhang et al., 2015). China is a major pecan consumer and needs to import around 50,000 tons of pecans per year to meet market demand, in addition to using its own domestic supply (Morales-de La Pena et al., 2023; Produce Report, 2024). Numerous epidemiological and clinical studies have documented the significant health benefits of pecans, including lowering blood cholesterol levels (Guarneiri et al., 2021) and preventing or improving cardiovascular disease (Atanasov et al., 2018) and diabetes (Yang, 2009). Lipids play a vital role in the quality and health benefits of pecans. Previous studies have reported that the oil content of pecans ranges from about 52.70 % to 78.07 % (Morales-de La Pena et al., 2023; Siebeneichler et al., 2023). Besides being consumed directly as a snack, pecans are a valuable source of high quality functional edible vegetable oils (Atanasov et al., 2018).

Pecan oil is noted for its high content of unsaturated fatty acids, which account for about 90 % of its total fatty acid content and more than 60 % of which are monounsaturated (Reis Ribeiro et al., 2020). These fatty acids play a crucial role in antioxidant activity, lowering plasma LDL cholesterol levels, and reducing the risk of chronic diseases (Atanasov et al., 2018; Yang, 2009). Additionally, studies have demonstrated that pecan oil contains high levels of squalene, phytosterols, and tocopherols (Atanasov et al., 2018; Miraliakbari & Shahidi, 2008), and that these compounds exhibit good antioxidant and anti-inflammatory properties (Oubannin et al., 2024). Previous researches have also indicated that pecan oil has a high smoke point (approximately 243 °C) (Maestri et al., 2020; Miraliakbari & Shahidi, 2008), making it suitable for high-temperature cooking. The growing emphasis on healthy consumption has driven increasing market demand for pecan oil as a high-quality vegetable oil. As the quality of vegetable oils is affected by the extraction method, it is necessary to assess the impact of different extraction methods on the quality of pecan oil (Maestri et al., 2020). This practice will facilitate the development of methods of producing pecan oil which meet the needs of consumers while being producer-friendly.

Traditionally, pecan oil is produced using mechanical pressing and solvent extraction (Alves et al., 2019; Maestri et al., 2020). The pressing method is favored for its simplicity, low cost, and ability to produce oil with superior color and flavor. However, this method suffers from poor extraction efficiency and is hampered by the need to perform extensive pretreatment of the raw materials (Costa-Singh & Jorge, 2015). In contrast, solvent extraction, which typically uses n-hexane, has higher extraction efficiency but entails risks of toxic chemical residues, environmental pollution and time-consuming (Zeng et al., 2024). The limitations of these traditional methods have prompted researchers and industry to explore new technologies to achieve efficient, cost-effective and environmentally friendly production of pecan oil (Maestri et al., 2020). An emerging technique, microwave-assisted solvent extraction, has been shown to significantly increase the efficiency of the extraction of tree nut oils (Maestri et al., 2020). Studies have demonstrated that this method not only reduces extraction time by approximately one-sixth but also improves the oil quality compared to traditional Soxhlet extraction (Hu et al., 2018; Maestri et al., 2020). Additionally, pressurized solvent extraction techniques, including supercritical and subcritical fluid extraction, have been applied to pecan oil extraction and align more closely with the concept of “green” extraction compared to traditional solvent methods (Alves et al., 2019; Scapinello et al., 2017). Supercritical fluid extraction utilizes solvents at temperatures and pressures above their critical points, while subcritical fluid extraction operates below these critical thresholds. Alves and colleagues optimized the process parameters for supercritical fluid extraction, achieving oil yields ranging from 52.26 % to 76.21 % (Alves et al., 2019). Furthermore, Scapinello et al. (2017) compared the efficiency of subcritical fluid extraction (using n-butane) with the cold-press method, finding that subcritical extraction (65 %, 40 bar) significantly outperformed cold-pressing (58.90 %) and produced oil with a higher 9c12cC18:2 n-6 content (26.20 %). Despite these advancements, current research on pecan oil extraction focuses mainly on optimizing parameters for individual methods, with emphasis on yield, fatty acid composition, and active compounds such as squalene and β-sitosterol (Alves et al., 2019; Costa-Singh & Jorge, 2015; Scapinello et al., 2017). However, comprehensive comparative studies on various extraction methods for pecan oil production remain limited (Fig. S1A). The aroma, which is a key factor in consumer acceptance and purchasing intent, is strongly influenced by the differences in the lipid molecules. Combining lipidomics and volatile metabolomics, as advanced methods of oil evaluation, can reveal changes at the molecular level of lipids and volatile compounds, and potentially reveal the relationship between lipids and aroma (Zeng et al., 2024). A review of the existing literature indicates that no studies have examined the effects of different extraction methods on the quality of pecan oil from the perspective of lipid molecules and aroma compounds using multi-omics techniques (Fig. S1B).

This study aimed to systematically assess the impact of conventional and non-conventional extraction methods on the lipid profiles and volatile compounds of pecan oil using lipidomics and volatile metabolomics. Additionally, it explored the intrinsic relationships between lipid molecules and aroma compounds under different extraction methods. The findings will provide a crucial theoretical foundation and practical guidance for environmentally friendly and consumer-oriented pecan oil production.

## 2 Materials and methods

### 2.1 Chemicals and regents

The chromatography grade acetonitrile, isopropanol, and methanol were purchased from CNW Technologies GmbH (Düsseldorf, Germany). Methyl tert-butyl ether (MTBE) and n-hexane were obtained from ANPEL Laboratory Technologies (Shanghai) Inc. (Shanghai, China). The ammonium formate was procured from Sinopharm Chemical Reagent Co., Ltd. (Shanghai, China). The potassium iodide and n-butane were acquired from Xilong Scientific Co., Ltd. (Shantou, China). The 14 % BF_3_-MeOH solution was obtained from Shanghai Aladdin Biochemical Technology Co., Ltd. (Shanghai, China). Dichloromethane, trichloromethane, acetic acid, and potassium hydroxide were purchased from Shanghai Macklin Biochemical Technology Co., Ltd. (Shanghai, China). The fatty acid methyl ester mixture standard (GLC-463) was purchased from NuChek Prep (Elysian, MN, USA).

### 2.2 Pecan fruit samples

Pecan fruits were harvested in October 2023 at the Jiangsu Long-term Research Base of Pecan Breeding and Cultivation (Luhe, Nanjing, China) (118.62°E, 32.48°N). The pecan variety used was Pawnee, which is one of the most widely grown varieties in China. After harvest, the pecan fruits were immediately transported to the laboratory and dried in an oven at 45 °C for 72 h. The dried pecans were then hand-shelled to obtain the kernels. The kernels were divided into three portions: one portion was retained as a sample, the other portion was used directly for oil extraction by pressing without grinding, and the third portion was ground into a powder and passed through a 50-mesh sieve to be used in other extraction methods. All samples were stored in sealed bags, shielded from light, and kept at −20 °C until analysis.

### 2.3 Extraction of oil from pecan

#### 2.3.1 Pressing extraction

The 500 g of pecan kernels were pressed in a small automatic screw-oil press (Model HANHUANG, Yueqing, China) to produce pecan oil. The temperature was kept at 70 ± 5 °C during the press and the speed was set at 30 rpm. The pecan oil obtained from pressing was filtered and centrifuged at 5000 rpm for 10 min to remove impurities, then transferred to brown bottles and stored in a − 20 °C refrigerator. This oil was labeled as PSE.

#### 2.3.2 Solvent extraction

Following the method of Gao et al. (2019), pecan oil was extracted using n-hexane as a solvent. Briefly, 10 g of pecan kernel powder was mixed with 100 mL of hexane in a conical flask wrapped in tin foil. The conical flasks were placed in an oscillator (YITONG, Jiangsu, China) and extracted by oscillation for 4 h. After filtering the mixture, the extract solution was gathered and rotary evaporated at 60 °C to eliminate the solvent, resulting in pecan oil with the designation STE.

#### 2.3.3 Microwave assisted solvent extraction

The pecan kernel powder (10 g) was first flattened in a microwave oven (Model NN-GF33KB, Panasonic, Osaka, Japan) and pre-treated at 700 W for 5 min. Subsequently, the solvent extraction procedure described in Section 2.3.2 was followed, but the extraction time was shortened to 2 h. Pecan oil extracted by microwave-assisted solvent extraction was labeled as MSE.

#### 2.3.4 Supercritical fluid extraction

The supercritical fluid extraction of pecan oil was conducted using a HA220–50-06 supercritical carbon dioxide extraction unit (Tongshi Hua'an Supercritical Extraction Co., Ltd., Jiangsu, China). The equipment consists of an extraction kettle, a separation kettle, a high-pressure CO_2_ pump, a CO_2_ storage tank, a cooling system, and a purification system. Maximum operating pressure is 50 MPa and maximum temperature 75 °C. Briefly, 500 g of pecan kernel powder was placed in the extraction kettle, and CO_2_ fluid in a supercritical state was pumped into the kettle at a flow rate of 30 L/h, with an extraction time of 4 h. Two sets of supercritical extraction experiments were conducted: the first with a pressure of 30 MPa and a temperature of 40 °C, and the second with a pressure of 35 MPa and a temperature of 45 °C (Zhang et al., 2018). After extraction, pecan oil was collected in a collecting vessel, weighed, transferred to a brown bottle, and stored at −20 °C, labeled SPFE30 (30 MPa, 40 °C) and SPFE35 (35 MPa, 45 °C).

#### 2.3.5 Subcritical fluid extraction

The subcritical extraction of pecan oil was performed using a CBE-5 L subcritical extraction unit provided by Henan Subcritical Biotechnology Co. (Henan, China). N-butane was selected as the solvent due to its superior molecular diffusion properties and faster mass transfer in the subcritical state (Scapinello et al., 2017). Briefly, 500 g of pecan kernel powder was placed in the extraction vessel, 2500 mL of n-butane was added, and extraction was carried out at 45 °C for 60 min, with a flow rate of 1 mL/min and pressure of 0.4 MPa. After two extraction cycles, the subcritical solvent was transferred to an evaporation system to remove the solvent. The resulting pecan oil was collected, weighed, transferred to a brown bottle, and stored at −20 °C for further analysis. The pecan oil obtained by subcritical fluid extraction was labeled SBFE.

### 2.4 Analysis of quality parameters

#### 2.4.1 Acid value and peroxide value

The acid value and peroxide value of pean oil were determined with reference to ISO 660. (2019) and ISO 3960. (2017), respectively.

#### 2.4.2 Oxidation stability index

The oxidation stability index (OSI) of pecan oil was determined using an 892 Professional Rancimat instrument (Metrohm, Switzerland). A sample of 3 g of pecan oil was placed in the measuring tube and analyzed at 120 °C with an air flow of 20 L/h. The results were expressed as an oxidation induction time period (h) (Gao et al., 2021).

### 2.5 Analysis of fatty acid composition

Fatty acid derivatization was performed following the BF_3_-methanol method (Ostermann et al., 2014). Specifically, 5 mg of the oil sample was mixed with 1.5 mL of 14 % BF_3_-methanol solution in a glass test tube, then shaken, mixed, and heated at 90 °C for 30 min. After cooling to room temperature, 1.5 mL of distilled water and 1.5 mL of n-hexane were added, mixed, and centrifuged at 3500 rpm for 5 min. The supernatant was collected, passed through a column of anhydrous sodium sulphate and dried under nitrogen to obtain a derivatized specimen.

The derivatized sample was then redissolved in 1 mL of hexane and transferred to a clean injection vial for gas chromatography-flame ionization detection (GC-FID) analysis. Fatty acid composition was analyzed using an Agilent 7890 A GC-FID system (Agilent Technologies, Santa Clara, CA) equipped with a CP-Sil 88 capillary column (100 m × 0.25 mm, 0.20 μm). The temperature of the autosampler and the detector was set at 250 °C. The injection volume was 1 μL with a split ratio of 10:1. The column oven temperature was initially set at 45 °C and held for 4 min, then increased to 175 °C at a rate of 13 °C/min and maintained for 27 min. Finally, the temperature was raised to 215 °C at a rate of 4 °C/min and held for 35 min. Fatty acids were characterized based on the retention times of reference standards, and quantitative analysis was performed using area normalization, with results presented as relative percentage area.

### 2.6 Lipidomics analysis

#### 2.6.1 Sample pretreatment

In this experiment, 10 mg of pecan oil was dissolved in 100 μL of dichloromethane-methanol solution (1:1, *v*/v) and thoroughly shaken. Subsequently, 30 μL of this mixture was combined with 970 μL of the dichloromethane-methanol solution (1:1, v/v) containing the internal standard in a clean injection vial, and the mixture was shaken to prepare the actual sample. Quality control (QC) samples were prepared by mixing 30 μL of supernatant from each sample in a different clean injection vial.

#### 2.6.2 UHPLC-MS/MS analysis

The lipid profiles of pecan oil were analyzed using a Vanquish ultra-high performance liquid chromatograph (UHPLC, Thermo Fisher Scientific, USA) coupled with an Orbitrap Exploris 120 mass spectrometer. Separation of lipid molecules was achieved on a Phenomenex Kinetex C18 liquid chromatography column (2.1 mm × 100 mm, 2.6 μm, Phenomenex, USA). The mobile phase was a biphasic system: the mobile phase A consisted of 10 % acetonitrile and 90 % isopropanol with 50 mL of a 10 mmol/L aqueous ammonium formate solution per 1000 mL of mobile phase A, and the mobile phase B consisted of 40 % water and 60 % acetonitrile with 10 mmol/L ammonium formate. The specific elution procedure was as follows: 0–1 min, 40 % A; 1–6.3 min, 40 %–85 % A; 6.3–8.6 min, 85 % A; 8.6–8.7 min, 85 %–100 % A; 8.7–9.3 min, 100 % A; 9.3–9.4 min, 100 % B-40 % A; 9.4–12 min, 40 % A. The column temperature was 55 °C.

Mass spectrometry (MS) data, encompassing both primary and secondary mass spectrometry information, were acquired using an Orbitrap Exploris 120 mass spectrometer operated via Xcalibur software (version 4.4, Thermo Fisher Scientific, USA). Data-dependent acquisition mode allowed for continuous evaluation of full-scan MS spectra. The specific parameters of the mass spectrometer were as follows: sheath gas flow rate of 30 Arb, auxiliary gas flow rate of 10 Arb, capillary temperature of 320 °C, full MS resolution of 60,000, MS/MS resolution of 15,000, collision energy of 15/30/45 in NCE mode, and spray voltage of 3.8 kV (positive ionization mode) or − 3.4 kV (negative ionization mode).

#### 2.6.3 Data preprocessing and annotation

The raw data files were converted to mzXML format using the msconvert function in ProteoWizard. The converted data were then processed with the R-based XCMS package. Specifically, the CentWave algorithm in XCMS was employed for peak detection, extraction, alignment, grouping, and integration. Lipid molecule annotation was achieved by matching spectral data with the LipidBlast library, developed in R and integrated with XCMS.

### 2.7 GC-IMS-based analysis of volatile compounds

Gas chromatography-ion mobility spectrometry (GC-IMS) analysis of volatile compounds was conducted using an Agilent 8090 gas chromatography system equipped with a G.A.S. ion mobility spectrometer and a PALRSL85 headspace automatic sampler. The volatile compounds were separated on an HP-5 capillary column (30 m × 0.32 mm, 0.25 μm film thickness, Agilent, USA). Briefly, 3 g of pecan oil was weighed accurately into a vial with a volume of 20 mL and incubated at 80 °C for 15 min with 500 rpm agitation. A headspace needle (85 °C) then aspirated 300 μL of the headspace gas for analysis. The column temperature was maintained at 60 °C. The ion mobility spectrometer was set to 45 °C. High-purity nitrogen was used as the drift and carrier gas, with a flow rate that started at 2 mL/min for 2 min, increased to 10 mL/min within 8 min, then increased to 100 mL/min within 10 min, and held at 100 mL/min for 10 min. The flow rate of drift gas was maintained at 150 mL/min.

Volatile compounds were identified by comparing retention indexes (RIs) and drift times with standards from the GC-IMS retrieval library (G.A.S., Dortmund, Germany). The volatile compound compositions of the different pecan oils were analyzed and visualized using VOCal software, including built-in plug-ins such as Reporter and GalleryPlot. The content of volatile compounds was expressed as a relative percentage. The relative odor activity value (ROAV) of each volatile compound was calculated according to the following formula (Zeng et al., 2024).

(1) $$\mathrm{ROA}V_{i}=\frac{C_{i}}{OT_{i}}\times \frac{{\mathrm{OT}}_{\max}}{C_{\max}}\times 100$$

where C_i_ is the relative percentage content of the compound, OT_i_ is the odor threshold of the compound, and C_max_ and OT_max_ are the relative percentage content of the component that contributes most to the overall aroma of the sample and the corresponding odor threshold, respectively. Compounds with a ROAV of more than 1 were considered to be the main volatile compounds in pecan oil.

### 2.8 Statistical analysis

All experiments were conducted in triplicate, and results were reported as mean ± standard deviation. Data were analyzed using IBM SPSS software (version 26, IBM, New York, USA). One-way analysis of variance (ANOVA) was performed to determine if there were statistically significant differences between group means, followed by Tukey's post hoc test for multiple comparisons. Statistical significance was set at *P* < 0.05. Visual analysis of the literature was conducted using CiteSpace software. The graphs and tables were generated using Origin 2021 (OriginLab, Northampton, MA, USA) and Microsoft PowerPoint (Microsoft, Washington, USA).

## 3 Results and discussion

### 3.1 Oil yield

As illustrated in Table 1, the yields of pecan oil varied significantly across different extraction methods, ranging from 56.01 % to 71.99 %. PSE (62.76) yielded significantly less oil than STE (70.13 %), in line with previous findings for oil from walnuts (Gao et al., 2019) and tiger nuts (Zhang et al., 2023). Excluding PSE, the yield ranking of the other methods was MSE (71.99 %) ≥ STE (70.13 %) ≥ SBFE (67.51 %) > SPFE35 (60.33 %) > SPFE30 (56.01 %). Compared to STE, MSE not only slightly increased oil yield but also halved the extraction time, demonstrating greater efficiency. This increase is probably due to absorption of high-frequency electromagnetic waves by the food matrix and water, which is rapidly converted to heat, resulting in an increase in intracellular pressure. This pressure disrupts the cell membranes and walls, facilitating solvent penetration and oil release (Hu et al., 2018). Similar findings have been observed in microwave-assisted avocado oil extraction (Moreno et al., 2003).

**Table 1:** The physicochemical properties and fatty acid composition of pecan oil extracted by different extraction methods.

	PSE	STE	MSE	SBFE	SPFE30	SPEF35
Oil yield (%)	62.76 ± 2.73^b^	70.13 ± 1.42^a^	71.99 ± 1.52^a^	67.51 ± 1.40^a^	56.01 ± 1.89^c^	60.33 ± 2.13^b^
Physicochemical properties						
Acid value (mg KOH/g)	0.68 ± 0.02^c^	0.41 ± 0.03^d^	0.68 ± 0.04^c^	0.32 ± 0.08^d^	1.43 ± 0.07^a^	1.16 ± 0.04^b^
Peroxide value (mmol/kg)	< 0.10^d^	< 0.10^d^	0.70 ± 0.11^c^	< 0.10^d^	1.52 ± 0.09^b^	1.69 ± 0.10^a^
Oxidative stability index (h)	7.91 ± 0.12^b^	7.59 ± 0.02^b^	10.96 ± 0.38^a^	7.87 ± 0.03^b^	5.73 ± 0.09^c^	4.95 ± 0.03^d^
Fatty acid (% total fatty acids)						
C12:0	0.15 ± 0.02^c^	0.51 ± 0.01^a^	0.23 ± 0.09^bc^	0.33 ± 0.07^b^	0.13 ± 0.07^c^	0.16 ± 0.09^c^
C14:0	0.49 ± 0.08^a^	0.52 ± 0.06^a^	0.50 ± 0.13^a^	0.52 ± 0.11^a^	0.47 ± 0.05^a^	0.39 ± 0.10^a^
C16:0	8.56 ± 0.09^d^	8.97 ± 0.25^cd^	10.36 ± 0.16^a^	9.20 ± 0.21^bc^	10.24 ± 0.07^a^	9.58 ± 0.31^bc^
9cC16:1	0.11 ± 0.04^a^	0.10 ± 0.04^a^	0.10 ± 0.04^a^	0.10 ± 0.04^a^	0.09 ± 0.00^a^	0.08 ± 0.00^a^
C17:0	0.09 ± 0.03^a^	0.12 ± 0.06^a^	0.16 ± 0.11^a^	0.11 ± 0.05^a^	0.08 ± 0.01^a^	0.10 ± 0.02^a^
10cC17:1	0.34 ± 0.22^a^	0.24 ± 0.03^a^	0.28 ± 0.07^a^	0.16 ± 0.06^a^	0.24 ± 0.02^a^	0.19 ± 0.01^a^
C18:0	3.42 ± 0.57^a^	2.96 ± 0.10^ab^	2.47 ± 0.03^bc^	2.52 ± 0.06^b^	2.40 ± 0.02^b^	2.63 ± 0.04^b^
9cC18:1	53.23 ± 1.09^ab^	54.03 ± 0.79^ab^	52.98 ± 0.49^ab^	54.86 ± 0.78^a^	52.16 ± 0.26^b^	54.16 ± 0.67^a^
11cC18:1	1.31 ± 0.38^a^	1.58 ± 0.22^a^	1.80 ± 0.13^a^	1.73 ± 0.07^a^	1.86 ± 0.10^a^	1.79 ± 0.17^a^
9c12cC18:2 n-6	29.52 ± 0.27^ab^	28.58 ± 0.57^bc^	28.55 ± 0.35^bc^	28.17 ± 0.57^c^	29.95 ± 0.25^a^	28.60 ± 0.58^bc^
C18:3 n-3	1.44 ± 0.03^a^	1.40 ± 0.01^bc^	1.35 ± 0.00^c^	1.43 ± 0.01^ab^	1.39 ± 0.01^bc^	1.38 ± 0.02^bc^
C20:2 n-6	0.41 ± 0.05^a^	0.33 ± 0.09^a^	0.44 ± 0.10^a^	0.25 ± 0.02^a^	0.32 ± 0.08^a^	0.33 ± 0.06^a^
C22:0	0.36 ± 0.14^a^	0.29 ± 0.08^a^	0.34 ± 0.11^a^	0.25 ± 0.02^a^	0.23 ± 0.01^a^	0.28 ± 0.04^a^
C20:4 n-6	0.35 ± 0.03^a^	0.30 ± 0.05^ab^	0.30 ± 0.07^ab^	0.26 ± 0.04^ab^	0.28 ± 0.01^ab^	0.24 ± 0.02^b^
C22:2 n-6	0.21 ± 0.14^a^	0.09 ± 0.06^a^	0.15 ± 0.01^a^	0.10 ± 0.01^a^	0.15 ± 0.02^a^	0.09 ± 0.03^a^
Total SFA	13.07 ± 0.76^b^	13.36 ± 0.12^ab^	14.06 ± 0.02^a^	12.93 ± 0.3^b^	13.56 ± 0.13^b^	13.13 ± 0.25^b^
Total MUFA	55.00 ± 0.59^bc^	55.94 ± 0.74^abc^	55.16 ± 0.53^bc^	56.86 ± 0.75^a^	54.35 ± 0.31^c^	56.22 ± 0.80^ab^
Total PUFA	31.93 ± 0.17^a^	30.70 ± 0.70^b^	30.78 ± 0.51^b^	30.21 ± 0.54^b^	32.09 ± 0.27^a^	30.65 ± 0.55^b^
Total EFA	31.31 ± 0.31^ab^	30.28 ± 0.57^bc^	30.20 ± 0.41^bc^	29.86 ± 0.54^c^	31.62 ± 0.24^a^	30.22 ± 0.57^bc^
AI	0.12	0.13	0.15	0.13	0.14	0.13
TI	0.26	0.27	0.29	0.26	0.28	0.27
H/H	9.26	8.48	7.55	8.47	7.77	8.38

Results were presented as mean ± standard deviation (*n* = 3). Different lowercase letters in the same row indicate significant differences between groups (*P* < 0.05), but groups that share the same letter do not differ significantly. AI, Atherogenicity index; EFA, essential fatty acids; H/H ratio, Hypocholesterolemic/hypercholesterolemic ratio; MUFA, monounsaturated fatty acid; PUFA, polyunsaturated fatty acids; SFA, saturated fatty acids; TI, Thrombogenicity index; UFA, unsaturated fatty acid. AI = [C12:0 + (4 × C14:0) + C16:0]/ΣUFA; TI = (C14:0 + C16:0 + C18:0)/[(0.5 × ΣMUFA) + (0.5 × Σn-6 PUFA) + (3 × Σn-3 PUFA) + (n-3 / n-6)]; HHI = (9cC18:1 + ΣPUFA)/(C12:0 + C14:0 + C16:0).

Interestingly, SBFE exhibited extraction efficiencies comparable to those of MSE and STE. In contrast, SPFE yielded significantly less oil than STE, MSE, and SBFE. However, a recent study on nut oil (Wen185) showed that the supercritical extraction method achieved the highest yield, exceeding both the subcritical liquid and solvent extraction methods (hexane as solvent) (Ma et al., 2024). This discrepancy suggests that extraction efficiency may vary depending on the matrix being studied. It is important to note that pressure and temperature are the critical factors influencing SPFE efficiency. Previous research has shown that in supercritical extraction systems, once the pressure reaches a critical point (the crossover point), the positive effect of the increased temperature-induced vapor pressure is outweighed by the negative effect of the decreased solvent density (Alves et al., 2019). In addition, the increased pressure increases the density of supercritical CO_2_, improving its solubility (Salvador et al., 2016). Salvador and colleagues examined the effects of varying supercritical pressure and temperature on pecan oil extraction efficiency, identifying the crossover point at approximately 300 bar (30 MPa) (Salvador et al., 2016). These findings likely explain why SPFE35 produced more oil than SPFE30 in this study.

### 3.2 Physicochemical properties

Table 1 presents the physicochemical parameters of pecan oil obtained by the different extraction methods. The acid value, which indicates the free fatty acid (FFA) content of the oil, serves as a measure of oil quality (Zhang et al., 2023). The results revealed that the acid values of SPFE30 (1.43 mg KOH/g) and SPFE35 (1.16 mg KOH/g) were significantly higher than those observed in the other extraction groups (0.32–0.68 mg KOH/g). One possible explanation is that small amounts of water in the matrix react with CO_2_ to form carbonic acid, or that supercritical CO_2_ preferentially dissolves the FFA, leading to an increase in the acid value of the product oil (Alves et al., 2019). Notably, the SBFE group had the lowest acid value, indicating higher oil quality. Even though the acid values varied, all of the results were less than those that had been previously documented for commercial pecan oil (1.70–1.90 mg KOH/g) (Salvador et al., 2016).

The peroxide value is a crucial indicator of hydroperoxide content formed during the early stages of oil oxidation, reflecting the degree of oxidative rancidity (Zhang et al., 2023). As shown in Table 1, the extraction method had a significant effect on the peroxide content of pecan oil, with the SPFE group showing significantly higher values (1.52–1.69 mmol/kg) than the other groups. It is worth noting that the instability of hydroperoxides and the formation of secondary oxidation products may account for the low peroxide values (< 0.10 mmol/kg) observed in PSE and STE oils (Gao et al., 2021). Overall, the peroxide values of pecan oil in this study were well below the Codex Alimentarius Commission recommended limit (< 10 mmol per kg) for edible vegetable oils.

The oxidation stability index of pecan oil, expressed as oxidation induction time (h), is also presented in Table 1. MSE group demonstrated the highest oxidative stability, with an induction time of 10.96 h. This finding is unexpected, as it is generally thought that heat treatment has a negative effect on nutritional compounds and natural antioxidants in foodstuffs (Zeng et al., 2024). However, the oil obtained through heat-treated extraction in this study exhibited strong oxidative stability, suggesting that antioxidant components may have been preserved from significant thermal degradation. Similar results were observed in a previous study where toasted pecans exhibited a higher antioxidant capacity than their unroasted counterparts (Kellett et al., 2019). Researchers speculated that heat treatment could release bound phenolics or produce Maillard reaction products (e.g., *Nigella sativa*), enhancing antioxidant activity (Kellett et al., 2019). Conversely, SPFE30 and SPFE35 groups exhibited poor oxidative stability, consistent with earlier studies on walnut oil, where the OSI values of oil extracted using supercritical fluids were significantly lower than those obtained via subcritical fluids and n-hexane (Gao et al., 2021; Ma et al., 2024). This can be attributed to the non-polar nature of compressed CO_2_, which makes it less efficient in the extraction of highly polarized antioxidants such as phospholipids and polyphenols (dos Santos et al., 2020). Additionally, the OSI values of pecan oil in this study were more than twice those of walnut oil (3.12–4.87 h) reported by Gao et al. (2021), indicating superior oxidative stability in pecan oil. In addition, a previous study comparing the oxidative stability of different nut oils (almonds, Brazil nuts, hazelnuts, pecans, pine nuts, pistachios, and walnuts) confirmed that pecan and pistachio oils are highly oxidative (Miraliakbari & Shahidi, 2008).

### 3.3 Fatty acid composition

The content of 15 fatty acids in pecan oil, ranging from C12:0 to C22:2 n-6, was analyzed and compared (Table 1). Among the fatty acid families, monounsaturated fatty acids (MUFA) were the most prevalent, comprising 54.35 % to 56.86 % of the total fatty acids, followed by polyunsaturated fatty acids (PUFA) at 30.21 % to 32.09 %, and saturated fatty acids (SFA) at 12.93 % to 14.06 %. Similarly, Siebeneichler et al. (2023) also noted that MUFA is dominant in pecan oil. However, studies of pecans in Texas and Brazil have reported that PUFA is the predominant family of fatty acids (Costa-Singh & Jorge, 2015; Yang, 2009), probably due to differences in cultivation practices (Siebeneichler et al., 2023). In this study, MSE oil had the highest SFA content (14.06 %), while the other groups did not show any significant differences in SFA content. The highest MUFA content (56.86 %) was found in the SBFE group, while the lowest MUFA content (54.35 %) was observed in the SPFE group. Regarding PUFA content, PSE (31.93 %) and SPFE30 (31.62 %) exhibited significantly higher levels than the other samples. Similar findings have been reported in studies on tiger nut oil (Zhang et al., 2023) and walnut oil (Gao et al., 2021), where the extraction method had minimal impact on fatty acid family composition, particularly SFA.

The primary SFA identified in pecan oil were C16:0 and C18:0 (Table 1), which aligns with the major SFA typically found in tree nut oils (Maestri et al., 2020). The highest concentration of C16:0 (10.36 %) was observed in MSE. Notably, the high PUFA content in pecan oil was largely due to the contribution of 9cC18:1 (Table 1). The content of 9cC18:1 ranged from 52.16 % (SPFE30) to 54.86 % (SBFE), accounting for nearly 96 % of the total MUFA. Previous researches have demonstrated that vegetable oils rich in 9cC18:1 exhibit superior antioxidant properties (Isleib et al., 2006; Maestri et al., 2020). Additionally, epidemiological studies support the preventive and ameliorative effects of 9cC18:1 on cardiovascular disease (Lu et al., 2024; Perdomo et al., 2015).

High levels of 9c12cC18:2 n-6 were detected in pecan oil, ranging from 28.58 % in the SBFE to 29.95 % in the SPFE30. A clinical cohort study indicated that high levels of 9c12cC18:2 n-6 are negatively associated with coronary heart disease mortality (Wu et al., 2014). C20:4 n-6, a precursor of pro-inflammatory eicosanoids, significantly influences the immune response (Wu et al., 2014). Low levels of C20:4 n-6, along with high levels of 9cC18:1 and 9c12cC18:2 n-6 in pecan oil, may help inhibit atherosclerosis. As displayed in Table 1, 9cC18:1 was consistently the predominant fatty acid in pecan oil, followed by 9c12cC18:2 n-6, regardless of the extraction method. This trend is consistent with previous findings on pecans harvested from the United States (Atanasov et al., 2018), Brazil (Siebeneichler et al., 2024), and Australia (Wakeling et al., 2001). However, a contrasting result was reported by Flores-Cordova, who found higher levels of 9c12c18:2 n-6 (47 %) in western Schley pecans from the Delicias region of Chihuahua, Mexico, compared with 9c18:1 (38.24 %) (Flores-Córdova et al., 2016). These findings suggest that the origin and variety of pecan may have a more significant impact on the 9cC18:1 and 9c12cC18:2 n-6 content of pecan oil than the extraction method.

To assess the impact of different extraction methods on the pecan oil fatty acid composition, we carried out a radial analysis of the fatty acid morphology (Fig. S2). Analysis showed that all the oil samples consisted mainly of MUFA, PUFA, 9c18:1 and 9c12c18:2 n-6. Although slight differences in the fatty acid content were observed between the different extraction methods, the overall trend was consistent. Several nutritional indices were introduced to evaluate the nutritional value of pecan oil fatty acids, including the essential fatty acids (EFA) content, atherogenicity index (AI), thrombogenicity index (TI), and hypocholesterolemic/hypercholesterolemic (H/H) ratio (Chen & Liu, 2020). EFA, primarily composed of 9c12cC18:2 n-6 and C18:3 n-3, is crucial for maintaining normal bodily functions and health. These fatty acids cannot be synthesized in the body and have to be obtained from food (Chen & Liu, 2020). Depending on the extraction method, the EFA content of pecan oil ranged from 29.86 % in SBFE to 31.62 % in SPFE30, indicating its high nutritional value. Both AI and TI reflect the relationship between fatty acids and the risk of atherosclerosis and thrombosis, respectively, with higher values indicating a greater risk. As shown in Table 1, the AI (0.12–0.15) and TI (0.26–0.29) values were low for all oil samples. Another study similarly reported lower values for AI (0.05–0.07) and TI (0.10–0.17) in oil extracted from pecan cake (dos Santos et al., 2020). The H/H ratio, which describes the relationship between fatty acid composition and the level of cholesterol, is inversely correlated with the risk of cardiovascular disease. In contrast to AI and TI, a higher H/H ratio suggests a lower risk. The H/H ratios of all analyzed pecan oils were high, ranging from 7.55 for MSE to 9.26 for PSE, suggesting that the consumption of pecan oil may reduce the incidence of cardiovascular disease.

### 3.4 Lipidomics analysis

#### 3.4.1 Overview of lipid molecules

A lipidomics approach based on UHPLC-MS/MS technology was employed to systematically analyze the effects of different extraction methods on the lipid composition of pecan oil (Hu et al., 2021). Total ion chromatograms (TIC) of the three QC samples demonstrated a strong overlap in both positive and negative ion modes (Fig. S3A). Similarly, the retention times and the peak areas of the internal standards in the QC samples exhibited a high consistency, as shown in the extracted ion chromatograms (Fig. S3B). These results indicated that the instrument-maintained stability during data acquisition. Furthermore, a correlation analysis of the QC samples confirmed the reliability and reproducibility of the experimental data (Fig. S3C).

**Fig. 1:**
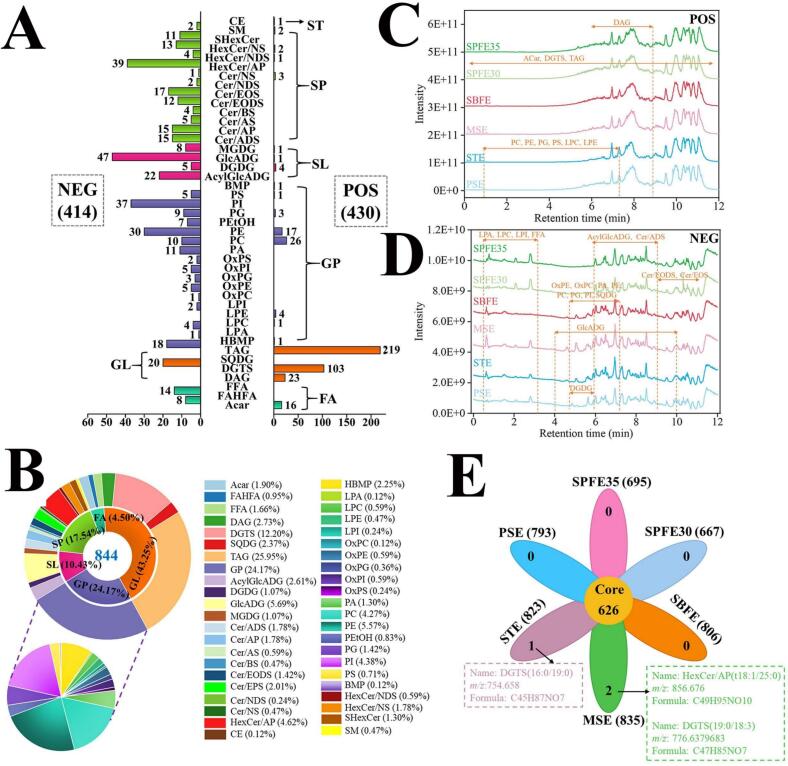
Overview of lipid molecules in pecan oil extracted by different extraction methods. Number of lipid molecules (A); Total ion chromatograms (TIC) of pecan oil in positive (B) and negative (C) ionization mode; Percentage of each category of lipids (D); Venn diagram of lipid molecules (E). POS, positive ionization mode; NEG, negative ionization mode.

In total, 844 lipid molecules were identified in pecan oil, with 430 detected in the positive ion mode, primarily as [M + H]^+^ and [M + NH4]^+^ ions. The remaining 414 lipid molecules were identified in the negative ion mode, mainly as [M-H]^−^ ions (Fig. 1A). Notably, acylcarnitine (ACar), diacylglycerol (DAG), and triacylglycerol (TAG) were detected exclusively in the positive ion mode, whereas FFA, fatty acid ester of hydroxyl fatty acid (FAHFA), sulfoquinovosyl diacylglycerol (SQDG), phosphatidic acid (PA), phosphatidylinositol (PI), acylglucuronosyldiacylglycerol (AcylGlcADG), ceramide alpha-hydroxy fatty acid-dihydrosphingosine (Cer/ADS), ceramide alpha-hydroxy fatty acid-phytospingosine (Cer/AP), ceramide alpha-hydroxy fatty acid-sphingosine (Cer/AS), ceramide beta-hydroxy fatty acid-sphingosine (Cer/BS), ceramide esterified omega-hydroxy fatty acid-dihydrosphingosine (Cer/EODS), ceramide esterified omega-hydroxy fatty acid-sphingosine (Cer/EOS), and ceramide non-hydroxyfatty acid-dihydrosphingosine (Cer/NDS) were identified exclusively in the negative ion mode. This detection mode was also observed in studies with hazelnut oil (Sun et al., 2022), *Camellia oleifera* oil (Zeng et al., 2024), and pecan embryos (Huang et al., 2022), where both TAG and DAG showed a good response in the positive ion mode, while PI showed a better response in the negative ion mode.

According to the lipid definition and classification strategy recommended by LIPID MAPS®, the 844 lipid molecules identified were classified into six major classes: fatty acyls (FA), glycerolipids (GL), glycerophospholipids (GP), saccharolipids (SL), sphingolipids (SP), and sterol lipids (ST). These classes were further subdivided into 43 lipid subclasses. GL were the most abundant in pecan oil, comprising 365 species (43.25 % of the total lipid count), followed by GP with 204 species (24.17 %) and SP with 148 species (17.54 %) (Fig. 1B). Within the GL class, triacylglycerol (TAG) was the predominant lipid subclass. The GP class consisted primarily of phosphatidylethanolamine (PE, 47 species, 5.57 %), phosphatidylcholine (PC, 36 species, 4.27 %), PI (37 species, 4.38 %), and HBMP (19 species, 2.25 %), and together they accounted for approximately 68.13 % of the total GP. This finding aligns with previous research where TAG and PE were identified as the major components of GL and GP, respectively, in *Camellia oleifera* seed oil (Zeng et al., 2024). Additionally, Hu et al. (2021) observed PC, PE, and PI as significant subclasses within the GP class in soybean, sesame, peanut, and rapeseed oils. However, unlike in the present study, PC was the predominant GP subclass in these oils (Hu et al., 2021).

A total of 148 SP molecules were characterized in pecan oil, with the HexCer/AP (39 species, 4.62 %) and Cer/EOS (17 species, 2.01 %) subclasses being the most dominant. The number of SP molecules identified in pecan oil was significantly higher than those previously reported in peanut oil (Liu et al., 2020), cactus fruit seed oil (Li et al., 2023), hazelnut oil (Sun et al., 2022), and *Camellia oleifera* oil (Zeng et al., 2024). Interestingly, 88 SL molecules were detected in pecan oil, accounting for 10.43 % of the total lipid count, with GlcADG (48 species, 5.69 %) and AcylGlcADG (22 species, 2.61 %) as the major subclasses. A recent study reported the presence of multiple SL molecules (51 species) during *Carya cathayensis* embryo development (Huang et al., 2022). Collectively, these data highlight the diversity and complexity of lipid molecules in pecan oil.

#### 3.4.2 Variations in lipids

As shown in Fig. 1C, D, the peak shape and the abundance of pecan oil TIC varied significantly depending on the type of extraction method used. The Venn diagram illustrates the number of lipid molecules and their overlap between different methods of lipid extraction (Fig. 1E). The highest lipid count was observed in MSE (835 species), while SPFE30 had the lowest (667 species). For example, HexCer (t18:1,25:0) and DGTS (t18:0,18:3) were detected only in the MSE group, whereas DGTS (16:0,18:3) was only found in the STE group.

**Fig. 2:**
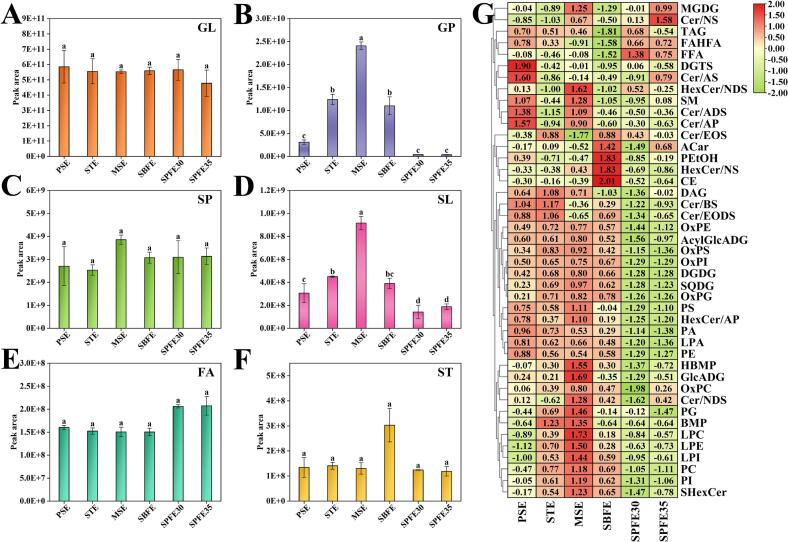
Variations in lipid content of pecan oil extracted by different extraction methods. Changes in the relative content of GL (A), GP (B), SP (C), SL (D), FA (E), and ST (F). Heat map analysis of lipid subclasses (G). Different lower-case letters for the same lipid class represent significant differences (*P* < 0.05). GL, glycerolipids; GP, glycerophospholipids; SP, sphingolipids; SL, saccharolipids; FA, fatty acyls; ST, sterol lipids.

To further explore the impact of different extraction methods on lipid levels, we aggregated the peak areas of each lipid class and presented the results as bar graphs (Fig. 2A-F). The findings did not reveal a statistically significant difference in GL and ST content between the groups, suggesting similar extraction capacity and efficacy of non-polar lipid methods. However, MSE showed higher extraction efficiency for the polar lipids GP and SL, while SPFE30 and SPFE35 were less efficient. The microwave treatment disrupts the cell membranes and walls, facilitating the extraction of GP and SL (Moreno et al., 2003). This result is in line with previous findings for oil extraction from *Camellia oleifera* where microwave-assisted solvent extraction resulted in the highest GP content (Zeng et al., 2024).

The poor performance of SPFE30 and SPFE35 in polar lipid extraction is mainly attributed to the low polarity of the solvent (Gao et al., 2019). CO_2_ in its supercritical state exhibits low polarity and is not suitable for the recovery of polar lipids (dos Santos et al., 2020). Notably, SBFE was second only to MSE in terms of efficiency of GP and SL extraction. The GP possess antioxidant properties that prolong the shelf life of the pecan oil and are also used as emulsifiers and stabilizers in the manufacture of foodstuffs and cosmetics (Liu et al., 2020). SL are involved in cell recognition and signaling, contribute to the functioning of the skin barrier, and are widely used in cosmetics and health foods (Huang et al., 2022).

The differences in relative abundance of the different lipid subclasses are shown in the heat map (Fig. 2G). The MSE group exhibited the highest levels of PC, PI, HBMP, PG, LPC, LPE, and LPI, reflecting the overall trend observed in total GP. The PC and PE are key to the development and functioning of the brain, and their amphiphilic structure makes them promising candidates for emulsion and drug delivery systems (Sun et al., 2022). GlcADG and total SL exhibited similar trends, with GlcADG predominating among the SL. GlcADG is a common component of plant membrane lipids; the microwave treatment significantly disrupted the biofilm, allowing for higher extraction levels of GlcADG in the MSE group. SPFE30 and SPFE35 showed a preference for FFA, likely due to carbon dioxide's selective extraction of FFA (Alves et al., 2019). Additionally, SBFE performed well in extracting ACar, PEtOH, HexCer/NS, and CE, while PSE was more effective in extracting DGTS and Cer/AS. DGTS, with its amphipathic properties similar to PC and PE, plays a crucial role in membrane lipid remodeling in lower green plants (Oishi et al., 2022). Furthermore, the distribution of major lipid subclasses in pecan oil (e.g., TAG, DGTS, PE, PC, PI, and GlcADG) closely mirrored their respective subclass distributions (Fig. S4 A-F).

#### 3.4.3 Multivariate statistical analysis based on lipidomics datasets

**Fig. 3:**
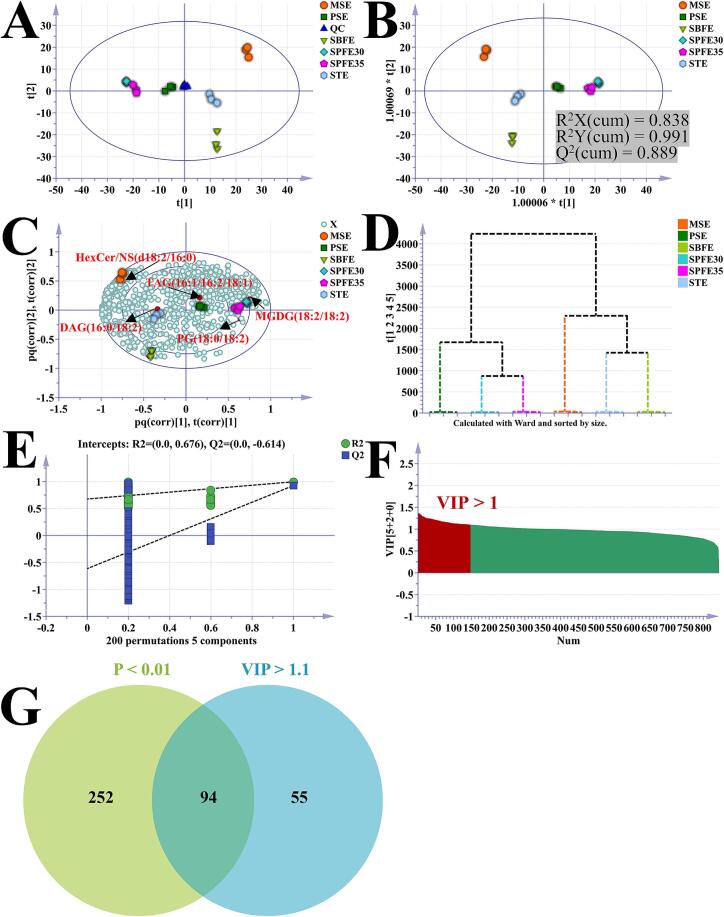
Multivariate statistical analysis of lipidomics data sets of pecan oil extracted by different extraction methods. 2D score plot for the PCA model (A); 2D score plot for the OPLS-DA model (B); Biplot for OPLS-DA model (C); Hierarchical cluster analysis diagram (D); 200 times permutation test result plot (E); Variable influence of projection (VIP) plot (F); Venn diagram of VIP and *P* value, lipid molecules meeting the criteria were differential lipids between pecan oils extracted by different methods (G).

Multivariate statistical analyses were carried out to further investigate the effects of the various extraction methods on the pecan oil lipid profile. An unsupervised principal component analysis (PCA) was initially performed to gain a preliminary understanding of the overall differences in the pecan oil samples from the various extraction methods. As shown in Fig. 3A, a significant separation was observed between all samples of pecan oil, indicating notable differences in their lipid profiles. Particularly notable was the tight clustering of the three QC samples, which confirmed the stability of the assay. Subsequently, the differences between the groups were analyzed using the orthogonal partial least squares-discriminant analysis (OPLS-DA) in supervised mode, allowing for the identification of differential lipid molecules. The OPLS-DA score plot (Fig. 3B) revealed a clear separation among the oil samples, with model fitness parameters of R^2^X(cum) = 0.838, R^2^Y(cum) = 0.991, and Q^2^ = 0.889, indicating strong explanatory and predictive capabilities. The OPLS-DA biplot (Fig. 3C) highlighted the contribution of each lipid molecule to the differentiation of pecan oil samples. Notably, DAG (16:0/18:2) and TAG (16:0/18:3/18:3) were predominant in the STE and SBFE groups, respectively, while HexCerNS (d18:2/16:0) was most prominent in the MSE group. Hierarchical cluster analysis (Fig. 3D) showed that MSE, STE, and SBFE formed one cluster, while PSE, SPFE30, and SPFE35 formed another cluster, indicating that the lipid composition of the SBFE, MSE, and STE groups are highly similar. Additionally, the 200-permutation test (Fig. 3E) confirmed that the OPLS-DA model had a reasonable fit and strong predictive ability. Based on variable influence of projection (VIP) value >1 and *P* value <0.01 criteria, 94 differential lipid molecules were identified, including 4 ACar, 2 AcylGlcADG, 13 Cer/ADS, 5 Cer/AP, 3 Cer/AS, 1 Cer/EODS, 6 Cer/EOS, 1 Cer/NDS, 1 Cer/NS, 12 DGTS, 2 FAHFA, 2 FFA, 5 GlcADG, 2 HBMP, 4 HexCer/AP, 1 HexCer/NS, 1 LPE, 7 MGDG, 2 OxPE, 1 PE, 1 PG, and 18 TAG (Fig. 3F-G and Table S1). These lipid molecules may serve as potential markers for distinguishing pecan oils obtained by different extraction techniques.

**Fig. 4:**
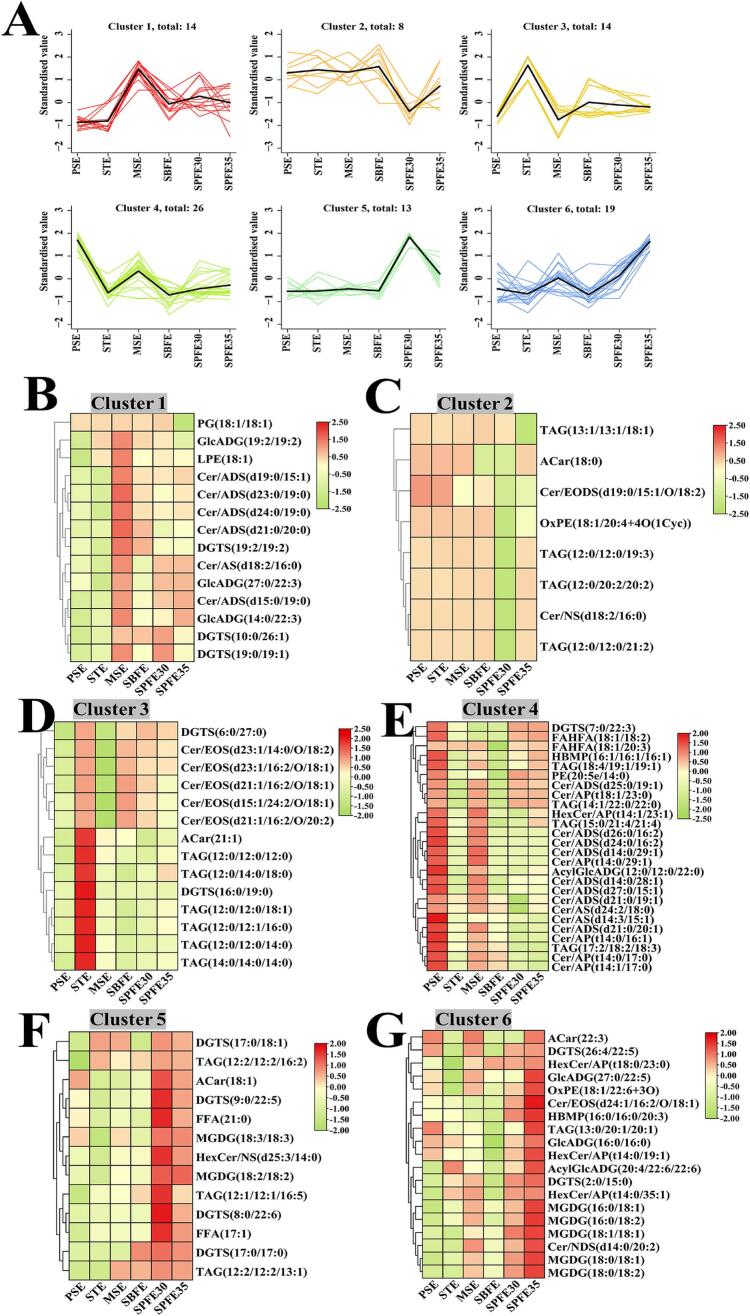
Variations in differential lipid molecules in pecan oil extracted different extraction methods. K-means cluster analysis of 94 differential lipid molecules (A). Visual analysis of heatmap clustering for Cluster 1–6 (B-G).

K-means clustering was conducted to further investigate changes in the relative abundance of the different lipid metabolites. The 94 differential lipid molecules were categorized into six major clusters based on their trends (Fig. 4A). Cluster 1 included 12 lipid molecules predominantly found in the MSE group, with the exceptions of DGTS (10:0/26:1) and PG (18:1/18:1) (Fig. 4B). Cluster 2 consisted of 8 lipid molecules, of which Cer/EODS (d19:0/15:1/O/18:2) and TAG (13:1/13:1/18:1) had the lowest levels in SPFE30 (Fig. 4C). Cluster 3 contained 14 lipid molecules, such as the TAG (12:0,12:0,12:0,18:1) and TAG (12:0,12:0,12:0,12:0) that were more common in the STE group (Fig. 4D). TAG (12:0/12:0/18:1) and TAG (12:0/12:0/12:0) have also been identified as major TAG molecules in basa catfish oil and coconut oil mixtures (Yuan et al., 2020). In Cluster 4 (Fig. 4E), FAHFA (18:1/18:2) and HBMP (16:1/16:1/16:1) levels were highest in the PSE group and lowest in the SBFE group. Clusters 5 and 6 contained 13 and 19 lipid molecules, respectively, predominantly found in the SPFE30 and SPFE35 groups (Fig. 4F, G). Notably, the MGDG molecules, including MGDG (16:0/18:1), MGDG (16:0/18:2), MGDG (18:0/18:1), MGDG (18:0/18:2), MGDG (18:1/18:1), MGDG (18:2/18:2), and MGDG (18:3/18:3), may be involved in membrane lipid metabolism (Song et al., 2023).

### 3.5 Volatile compound analysis based on GC-IMS

#### 3.5.1 Changes in volatile compounds

The aroma of the vegetable oil is a critical factor influencing its quality. Therefore, this study employed the GC-IMS to investigate the aroma profile of pecan oil obtained through various extraction methods. As illustrated in the 3D-topography plot (Fig. S5 A), although the volatile compound composition across the six pecan oil groups was similar, significant differences were observed in the intensity of the component peaks. To better visualize the signal intensity of the volatile compounds, a top view of the 3D-topography plot was generated (Fig. S6A). In this graph, each dot represents a volatile compound, and the red dot indicates the higher intensity of the signal. Most signals appeared between 0 and 1000 s with a drift time of 1.0 to 1.5 s (relative to RIP). Additionally, a 2D comparative topography plot (Fig. S6B) was created by using the volatile compound content in the PSE group as a reference and subtracting this value from the content in the other groups. The plot uses different colors to indicate the differences in the volatile compound intensity between the groups: white indicates a similar intensity, blue indicates a lower intensity and red indicates a higher intensity. The results clearly demonstrated that the aroma profile of pecan oil varies significantly depending on the extraction method.

**Fig. 5:**
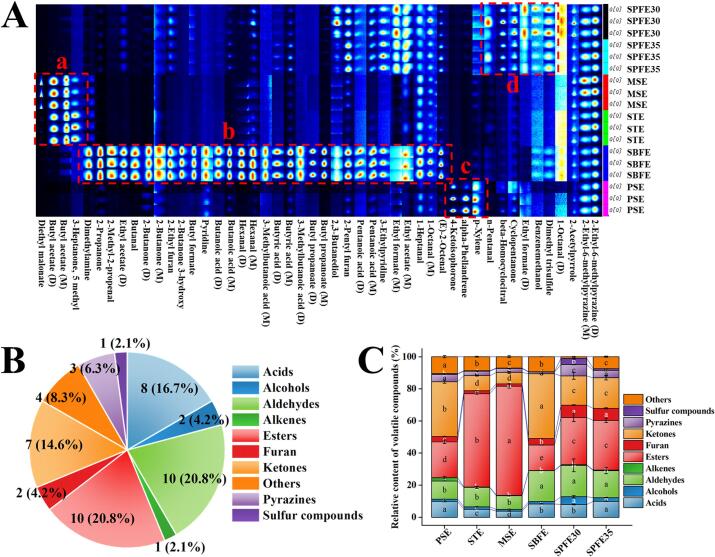
Overview of volatile compounds of pecan oil extracted by different extraction methods. Gallery plot comparison of volatile compounds (A); Number and percentage of each category of volatile compound (B). Changes in the relative content of volatile compound categories (C).

The topographic map with markers (Fig. S5B) provided a qualitative analysis of volatile compounds, identifying 48 distinct compounds. Detailed information on these compounds is given in Table S2. The carbon chain lengths of these volatile compounds range from C4 to C11, with some compounds, such as 1-octanal and hexanal, exhibiting bimodal peaks for monomers and dimers. This bimodal distribution is acceptable, since compounds with high proton affinity may form a dimer in the presence of drift (Zheng et al., 2024). Subsequently, using the Gallery Plot plug-in, fingerprints of these 48 volatile compounds were automatically generated (Fig. 5A). In the gallery plot, the brightness of each substance corresponds to its abundance. Four distinct regions (a, b, c, and d) were highlighted in the gallery plot. In the region a, 5-methyl-3-heptanone, butyl acetate (D) and butyl acetate (M) were found at relatively high levels in the MSE and STE groups. In region b, SBFE oil contained elevated levels of 31 volatile compounds, including (E)-2-octenal, 1-heptanal, 1-octanal (M), 2-methyl-2-propenal, and 2,3-butanediol. Hexanal, which gives off a cycad or green smell, is a breakdown product of 9c12c18:2 n-6 and may be positively correlated with 9c12c18:2 n-6, as reported in earlier studies with pecans (Reis Ribeiro et al., 2020) and walnuts (Torres et al., 2005). Additionally, previous studies have detected 2-methyl propanol, a precursor to 2-methyl-2-propenal, in pecans (Gong et al., 2018; Reis Ribeiro et al., 2020). As regards region c, p-xylene, alpha-phellandrene, and 4-ketoisophorone were significantly more concentrated in the PSE group than in the other groups. Conversely, region d exhibited high levels of dimethyl trisulfide, benzenemethanol, ethyl formate (D), cyclopentanone, beta-homocyclocitral, and n-pentanal in the SPFE30 and SPFE35 groups. Cyclopentanone, known for its caramel aroma, is commonly found in roasted sesame oil (Yin et al., 2021) and *Camellia oleifera* oil (Zeng et al., 2024). Notably, 2-ethyl-6-methylpyrazine (D) and 2-ethyl-6-methylpyrazine (M), which are pyrazine derivatives with a nutty aroma, were detected at high levels in all pecan oil samples.

The 48 identified volatile compounds were further classified, revealing that the predominant volatile compounds in pecan oil were aldehydes and esters (Fig. 5B). The percentages of volatile compound categories in pecan oil varied significantly with different extraction methods, with the highest proportions of esters in the MSE and STE groups (Fig. 5C). Esters with lower excretion thresholds had a significant effect on the taste profiles of pecan oil (Table 2). A recent study reported a higher ester content in *Camellia oleifera* oil extracted with n-hexane, which is consistent with the findings of this study (Zeng et al., 2024). It is noteworthy that increasing microwave time may elevate the proportion of aldehydes in the oil (He et al., 2021). The highest proportion of aldehydes was observed in groups SPFE30 and SPFE35, while the highest ketone content was found in groups SBFE and PSE. Aldehydes and ketones typically arise from the oxidative degradation of fatty acids or the Maillard reaction. The higher proportion of these compounds in these four groups suggested a weaker antioxidant capacity, consistent with the OSI results discussed earlier (Table 1). Similarly, high levels of aldehydes and ketones have been reported in the camellia seed oil obtained by pressing method (Zeng et al., 2024). Previous research has identified alcohols and acids as the most abundant volatile compounds in pecan powder (Reis Ribeiro et al., 2020), contrasting with the current findings, where esters and aldehydes predominated in pecan oil. It is hypothesized that chemical reactions such as esterification and oxidation of alcohols and acids during oil extraction may have produced esters (fruity and floral) and aldehydes (fatty, grassy, and fruity). Although fewer aldehydes were detected in pecan oil (10 species) than in pecan powder (12 species), 1-octanal, 1-heptanal, 2-methyl-2-propenal, and hexanal were found in both, suggesting these aldehydes play a key role in the aroma of the products derived from pecans (Reis Ribeiro et al., 2020). Overall, the extraction methods significantly influenced the aromatic profiles of the pecan oil.

**Table 2:** The effect of different extraction methods on main volatile compounds (ROAV >1) of pecan oil.

Compounds	Category	Threshold (mg/kg)	PSE		STE		MSE		SBFE		SPFE30		SPFE35	
			ROAV	Relative content (%)	ROAV	Relative content (%)	ROAV	Relative content (%)	ROAV	Relative content (%)	ROAV	Relative content (%)	ROAV	Relative content (%)
Butanoic acid (M)	Acids	0.05	0.92	2.12 ± 0.18	0.59	0.85 ± 0.12	0.64	0.71 ± 0.05	2.40	2.34 ± 0.07	0.30	1.58 ± 0.21	0.36	1.99 ± 0.09
Butyric acid (D)	Acids	0.0015	10.63	0.73 ± 0.17	9.19	0.40 ± 0.01	8.50	0.28 ± 0.02	24.98	0.73 ± 0.02	3.50	0.55 ± 0.06	4.87	0.81 ± 0.07
Butyric acid (M)	Acids	0.0015	34.40	2.37 ± 0.31	25.44	1.10 ± 0.01	26.23	0.87 ± 0.10	64.70	1.89 ± 0.03	9.96	1.56 ± 0.02	15.81	2.63 ± 0.11
(E)-2-Octenal	Aldehydes	0.003	9.40	1.30 ± 0.16	10.79	0.93 ± 0.08	8.13	0.54 ± 0.10	16.61	0.97 ± 0.04	4.44	1.39 ± 0.05	7.07	2.35 ± 0.15
1-Heptanal	Aldehydes	0.0028	8.08	1.04 ± 0.10	8.48	0.68 ± 0.11	10.69	0.66 ± 0.06	8.02	0.44 ± 0.01	3.63	1.06 ± 0.04	3.20	0.99 ± 0.12
1-Octanal (D)	Aldehydes	0.0007	5.64	0.18 ± 0.02	5.36	0.11 ± 0.02	5.75	0.09 ± 0.01	4.48	0.06 ± 0.01	2.74	0.20 ± 0.02	2.41	0.19 ± 0.01
1-Octanal (M)	Aldehydes	0.0007	35.65	1.15 ± 0.10	38.93	0.79 ± 0.05	44.66	0.69 ± 0.02	30.55	0.42 ± 0.01	19.14	1.4 ± 0.09	16.63	1.29 ± 0.06
2-Methyl-2-propenal	Aldehydes	0.025	0.26	0.30 ± 0.03	0.26	0.18 ± 0.01	0.22	0.12 ± 0.01	2.92	1.42 ± 0.03	0.10	0.26 ± 0.04	0.11	0.30 ± 0.06
Butanal	Aldehydes	0.009	2.15	0.89 ± 0.04	1.68	0.44 ± 0.04	1.85	0.37 ± 0.03	21.58	3.78 ± 0.05	0.97	0.91 ± 0.05	1.08	1.07 ± 0.18
Hexanal (D)	Aldehydes	0.0045	8.25	1.71 ± 0.07	24.21	3.14 ± 0.06	25.02	2.50 ± 0.07	70.05	6.14 ± 0.04	4.62	2.17 ± 0.59	3.25	1.62 ± 0.07
Hexanal (M)	Aldehydes	0.0045	13.60	2.81 ± 0.15	34.50	4.48 ± 0.10	26.65	2.66 ± 0.12	63.08	5.53 ± 0.05	12.09	5.69 ± 1.13	6.62	3.30 ± 0.24
n-Pentanal	Aldehydes	0.012	2.18	1.20 ± 0.12	3.08	1.07 ± 0.41	2.48	0.66 ± 0.03	1.67	0.39 ± 0.03	4.44	5.57 ± 2.33	3.62	4.82 ± 1.59
alpha-Phellandrene	Alkenes	0.006	7.56	2.09 ± 0.77	1.31	0.23 ± 0.03	1.43	0.19 ± 0.00	0.69	0.08 ± 0.01	0.57	0.36 ± 0.05	0.57	0.38 ± 0.04
Butyl acetate (D)	Esters	0.66	0.05	1.52 ± 0.03	1.48	28.13 ± 0.14	1.89	27.69 ± 0.97	0.04	0.47 ± 0.03	0.02	1.26 ± 0.21	0.02	1.29 ± 0.11
Butyl propanoate (D)	Esters	0.025	1.53	1.76 ± 0.12	1.50	1.08 ± 0.06	1.44	0.80 ± 0.09	2.81	1.37 ± 0.02	0.52	1.35 ± 0.15	0.49	1.37 ± 0.04
Butyl propanoate (M)	Esters	0.025	1.54	1.77 ± 0.22	1.51	1.09 ± 0.11	1.49	0.83 ± 0.07	7.76	3.78 ± 0.03	0.75	1.95 ± 0.27	0.60	1.66 ± 0.07
Diethyl malonate	Esters	0.012	13.25	7.31 ± 0.77	15.39	5.33 ± 0.32	79.59	21.2 ± 2.73	6.92	1.62 ± 0.14	4.04	5.06 ± 0.85	4.55	6.05 ± 0.29
Ethyl acetate (D)	Esters	0.005	4.20	0.97 ± 0.08	4.01	0.58 ± 0.03	3.95	0.44 ± 0.02	34.71	3.38 ± 0.03	4.83	2.53 ± 0.31	5.48	3.04 ± 0.16
Ethyl acetate (M)	Esters	0.005	6.10	1.40 ± 0.10	7.27	1.05 ± 0.49	9.10	1.01 ± 0.35	11.84	1.15 ± 0.03	9.95	5.20 ± 0.43	11.88	6.58 ± 0.32
2-Pentyl furan	Furan	0.0058	3.60	0.96 ± 0.17	2.72	0.45 ± 0.06	3.82	0.49 ± 0.01	6.92	0.78 ± 0.03	3.17	1.92 ± 0.05	4.39	2.82 ± 0.16
2-Butanone (D)	Ketones	0.014	8.35	5.37 ± 0.48	1.82	0.74 ± 0.04	1.88	0.58 ± 0.07	20.04	5.46 ± 0.02	1.34	1.96 ± 0.27	1.08	1.68 ± 0.11
2-Butanone (M)	Ketones	0.014	4.71	3.03 ± 0.50	4.23	1.71 ± 0.31	3.56	1.11 ± 0.06	7.14	1.95 ± 0.03	1.52	2.22 ± 0.04	1.45	2.24 ± 0.13
2-Butanone 3-hydroxy	Ketones	0.055	0.88	2.21 ± 0.16	0.88	1.40 ± 0.06	0.92	1.12 ± 0.20	2.06	2.21 ± 0.05	0.26	1.47 ± 0.12	0.29	1.79 ± 0.19
Dimethyl trisulfide	Sulfur compounds	0.0001	100	0.46 ± 0.08	100	0.29 ± 0.02	100	0.22 ± 0.02	100	0.19 ± 0.01	100	1.05 ± 0.14	100	1.11 ± 0.03

The odor thresholds of volatile compound were obtained from the book named “COMPILATIONS OF ODOR THRESHOLD VALUES IN AIR, WATER AND OTHER MEDIA (SECOND ENLARGED AND REVISED EDITION)”.

#### 3.5.2 Multivariate statistical analysis based on volatile compound datasets

**Fig. 6:**
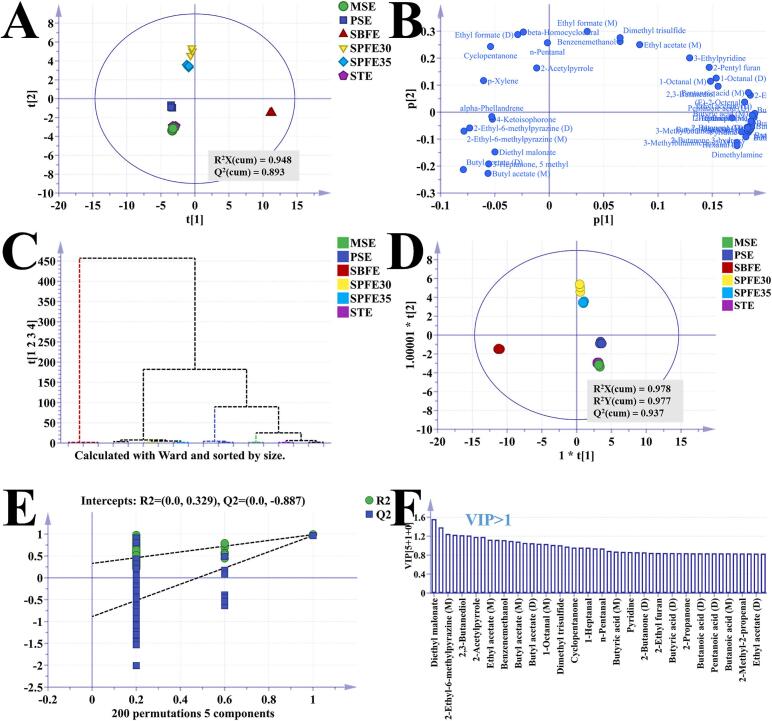
Multivariate statistical analysis of volatile compounds in pecan oil extracted by different extraction methods. 2D score plot for the PCA model (A); Loading plot of the PCA model (B); Hierarchical cluster analysis (C); 2D score plot for the OPLS-DA model (D); 200 times permutation test result plot (E); Variable influence of projection (VIP) plot (F).

Fig. 6A presents the score plot of the PCA model based on the GC-IMS dataset, where significant differentiation between pecan oil sample groups was evident. Of particular note, the close clustering of samples in the MSE and STE groups suggested a high degree of similarity in their volatile compound compositions. Principal components 1 and 2 of the PCA model accounted for 58.10 % and 21.80 % of the total variance, respectively. The model demonstrated a cumulative R^2^X of 0.948 and a Q^2^ of 0.893, indicating that the principal components effectively captured the variation in the initial variables. Butyl acetate (M) and butyl acetate (D) were the key volatile compounds in the PSE and MSE groups, as shown in Fig. 6B. Hexanal (D) was one of the main contributors distinguishing the SBFE group from the others. Hierarchical cluster analysis revealed that the SBFE group had significantly different volatile compound profiles from the other five groups (Fig. 6C). MSE, PSE, and STE clustered together, while SPFE30 and SPFE35 formed another cluster, reflecting the similarity in volatile compound profiles among these samples. The OPLS-DA model score plot exhibited similar distribution trends to the PCA score plot, with R^2^X (cum) = 0.978, R^2^Y (cum) = 0.977, and Q^2^ = 0.937 (Fig. 6D). The 200-permutation test (R^2^ = 0.329, Q^2^ = −0.887) further validated the robustness and predictive capability of the model (Fig. 6E).

In addition to VIP and *P* values, the ROAV was used to assess the contribution of each volatile compound to the overall aroma. Table 2 lists the relative percentage content of volatile compounds with ROAV values greater than 1. Dimethyl trisulfide, a volatile compound with a minimal odor threshold, contributed the most to the overall flavor of all the pecan oil samples. Disregarding dimethyl trisulfide, aldehydes and acids were the largest contributors to the overall odor in both PSE and STE oils. In MSE, esters and aldehydes were the primary contributors, while aldehydes were predominant in the SPFE30 and SPFE35 oils. Interestingly, in the SBFE group, acids, aldehydes, esters, and ketones all had a higher ROAV value (> 20), suggesting that the oil samples in this group had a more complex aroma profile.

**Fig. 7:**
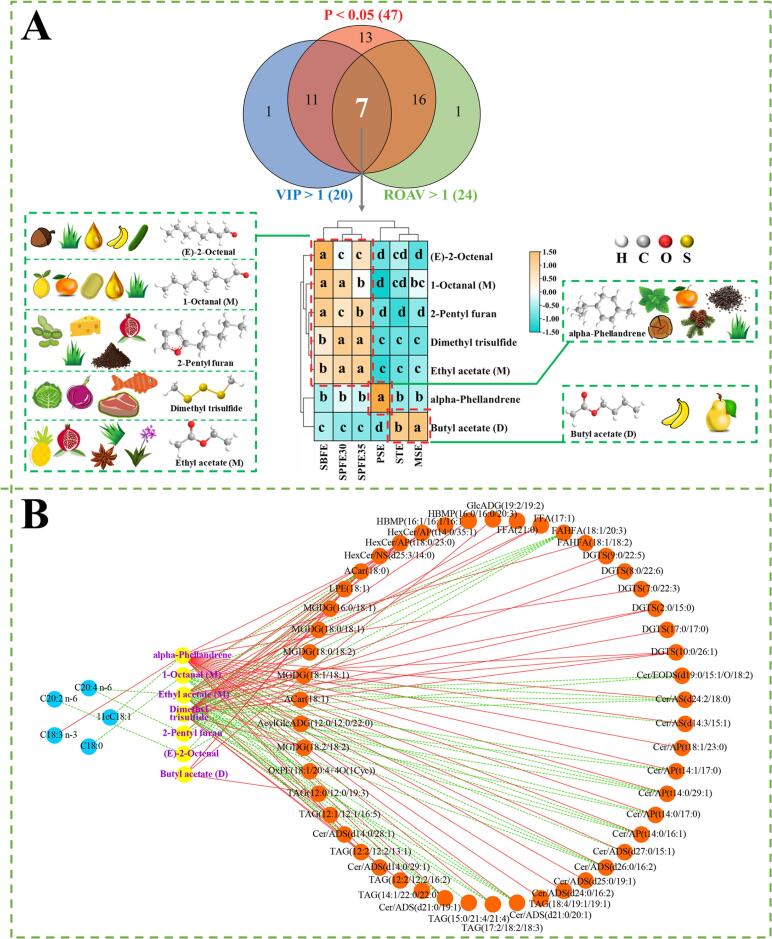
Differential metabolite screening and correlation analysis. The heatmap of seven key differentially volatile compounds (VIP > 1, *P* < 0.01, and ROAV >1) (A). Pearson correlation network analysis between fatty acids, differential lipid molecules, and key volatile compounds (B). In the heat map, the color-coded scale from red to green corresponds to a change in the content of volatile compounds from high to low. Different letters in the same row represent significant differences (*P* < 0.05). In the correlation network diagram, the red solid line represents positive correlation and the green dashed line represents negative correlation. (For interpretation of the references to color in this figure legend, the reader is referred to the web version of this article.)

The criteria for the identification of key volatile compounds were set at VIP > 1, *P* < 0.05, and ROAV >1. Based on these criteria, 7 key volatile compounds were identified, including 2 aldehydes, 2 esters, 1 alkene, 1 furan, and 1 sulfur compound (Fig. 6F and Fig. 7A). Among these, (E)-2-octenal, 1-octanal (M), and 2-pentyl furan were predominantly found in SBFE oil, whereas the SPFE30 and SPFE35 oils had the highest levels of dimethyl trisulfide and ethyl acetate (M). Ethyl acetate was previously reported to be an important ester in pecan (Reis Ribeiro et al., 2020). Contrary to the findings of this study, however, Zeng et al. (2024) stated that the content of ethyl acetate (M) in *Camellia oleifera* oil obtained by the Soxhlet method (using n-hexane as solvent) was higher than in the oil samples obtained by the pressing and supercritical fluid methods. This discrepancy may be attributed to the varying effects of volatile compounds on different plant matrices under similar extraction methods. Butyl acetate (D) was prominent in the STE and MSE groups, while alpha-phellandrene was more abundant in the PSE group.

### 3.6 Correlation analysis

The formation of volatile compounds in vegetable oils, such as alcohols, esters, aldehydes and ketones, is closely related to the lipid composition, since lipid molecules are often used as a precursor to aromatic compounds. Pearson correlation coefficients (|r| > 0.6) were used to visualize the correlations between fatty acids, key volatile compounds, and differential lipid molecules, as depicted in Fig. 7B. In the correlation network diagram, the green dashed line indicates a negative correlation, while red solid line represents a positive correlation. Lipids exhibited complex correlations with key volatile compounds. For example, C18:3 n-3 showed a positive correlation with alpha-phellandrene, whereas 11cC18:1 showed a negative correlation with alpha-phellandrene. In addition, most lipid molecules correlated positively with alpha-phellandrenes, indicating that oxidative decarboxylation of lipids is a potential route to formation of alkene (Yin et al., 2021). Further analysis revealed that ACar (18:0), C20:2 n-6, and FAHFA (18:1/20:3) were negatively correlated with (E)-2-octenal. FAHFA (18:1/20:3) also exhibited a strong negative correlation with 1-octanal (M). Under heating conditions, 9cC18:1 generates 1-octanal (M) via the ROOH pathway starting at the C11 site (Torres et al., 2005). Additionally, the MGDG, TAG, and DGTS lipid molecules mostly displayed a negative correlation with ethyl acetate (M), whereas ACar (18:0), GlcADG (19:2/19:2), LPE (18:1), and TAG (12:0/12:0/19:3) showed a positive correlation with butyl acetate (D). It has been suggested that ethyl acetate and butyl acetate may be formed by esterification of ethanol, produced through β-oxidation of acetic acid, with the corresponding alcohols (ethanol and butanol) (Zeng et al., 2024), and that both compounds typically have a fruity odor (He et al., 2021). Moreover, ACar (18:0), C20:4 n-6, and FAHFA (18:1/20:3) showed a positive correlation with 2-pentyl furan, while HexCer/AP (t18:0/23:0) had a negative correlation with 2-pentyl furan. 2-Pentyl furan is prevalent in heat-processed foods and may be a product of 9c12cC18:2 n-6 oxidation (Yin et al., 2024).

## 4 Conclusion

In conclusion, the choice of the extraction method had a significant impact on the efficiency and quality of the extraction of pecan oil. MSE (71.99 %) and SBFE (67.51) achieved oil yields comparable to those obtained by STE (70.13) but required shorter extraction times and a smaller solvent content. SPFE method increased the acid value of oil. MSE provided the highest oxidative stability (10.96 h), whereas SPFE demonstrated poor antioxidant stability. Pecan oil was rich in oleic and linoleic acids and had a low AI and TI and a high H/H ratio. Although the various extraction methods had a minor influence on the fatty acid composition, they had a significant effect on the lipid molecular profiles. In terms of GP and SL extraction, MSE was the most efficient, followed by SBFE, and SPFE was the least efficient. Ninety-four differential lipids were identified as potential markers. In addition, 48 volatile compounds were detected, with aldehydes and esters as the dominant categories. Dimethyl trisulfide was the most significant contributor to the overall aroma of pecan oil. Seven key volatile compounds were identified, including α-phellandrene, (E)-2-octenal, 1-octanal, 2-pentyl furan, dimethyl trisulfide, ethyl acetate, and butyl acetate. Correlation analysis showed that the lipid changes due to the different extraction methods resulted in changes in the aromatic characteristics of the pecan oil. These findings suggest that microwave-assisted solvent extraction and subcritical extraction are highly efficient and environmentally sustainable alternatives to traditional mechanical press and solvent extraction methods.

## CRediT authorship contribution statement

**Jingtao Cui:** Writing – review & editing, Writing – original draft, Visualization, Methodology, Investigation. **Li Cui:** Writing – original draft, Investigation. **Tao Zhang:** Validation, Software. **Xinchen Jiang:** Formal analysis. **Yaming Qian:** Investigation. **Wuyang Huang:** Writing – review & editing, Resources, Funding acquisition. **Haijun Zhu:** Writing – review & editing, Supervision, Funding acquisition, Conceptualization.

## Declaration of competing interest

The authors declare that they have no known competing financial interests or personal relationships that could have influenced the work reported in this paper.

## Data Availability

Data will be made available on request.

## Appendix A Supplementary data

Supplementary material: Fig. S1.-Fig. S7. Image 1
