# Supplementary material for: Comparative analysis of lipid profiles and aroma characteristics in pecan oils: Probing conventional and non-conventional extraction techniques
Source: Food Chem X. 2025 Apr 16;27:102470. doi: 10.1016/j.fochx.2025.102470 (PMC12032943; doi:10.1016/j.fochx.2025.102470)
Supplement: Supplementary file 1 — Supplementary material: Fig. S1.-Fig. S7. [file mmc1.docx]

**Figure Captions**

**Fig. S1**. Literature visualization and analysis of pecan oil. Retrieve keywords (pecan oil, pecan lipid, *Carya illinoensis* (Wangh.) Koch oil, and *Carya illinoensis* (Wangh.) Koch lipid) in the Web of Science Core Collection database. Overlay visualization of the Keywords in selected 243 references from 2009 to 2024. Keyword co-occurrence map (A). Key words Cluster map (B).

**Fig. S2.** Morphotypes of fatty acids from pecan oils extracted by different methods.

**Fig. S3.** Reliability analysis of lipidomics procedure. Total ion chromatograms (TIC) of quality control samples (QC) (A); The extracted ion chromatograms (EIC) plot of internal standard substances in QC samples (B); Correlation analysis of three QC samples (C).

**Fig. S4.** Variations in the content of TAG (A), DGTS (B), PE (C), PC (D), PI (E), GlcADG (F) molecules in pecan oil extracted by different extraction methods. TAG, triacylglycerol; DGTS, diacylglyceryl trimethylhomoserine; PE, phosphatidylethanolamine; PC, phosphatidylcholine; PI, phosphatidylinositol; GlcADG, glucuronosyldiacylglycerol.

**Fig. S5.** Volatile compound analysis based on GC-IMS. 3D topographic plot of volatile compound profiles of pecan oils extracted by different methods (A). Topographic plots of GC-IMS spectra with individual volatile compound identification marked 1–48 (B).

**Fig. S6.** GC-IMS spectrogram of pecan oil extracted by different extraction methods. The two-dimensional topographic plot (A). Comparison of the difference spectrum of volatile compounds (B). With PSE as the reference, the signal peak in PSE is subtracted from the rest of the spectra and the difference in the spectra of the two samples was obtained. The blue color means that the substance is lower than PSE and the red dot means that the substance is higher than PSE.





**Fig. S1.**

**
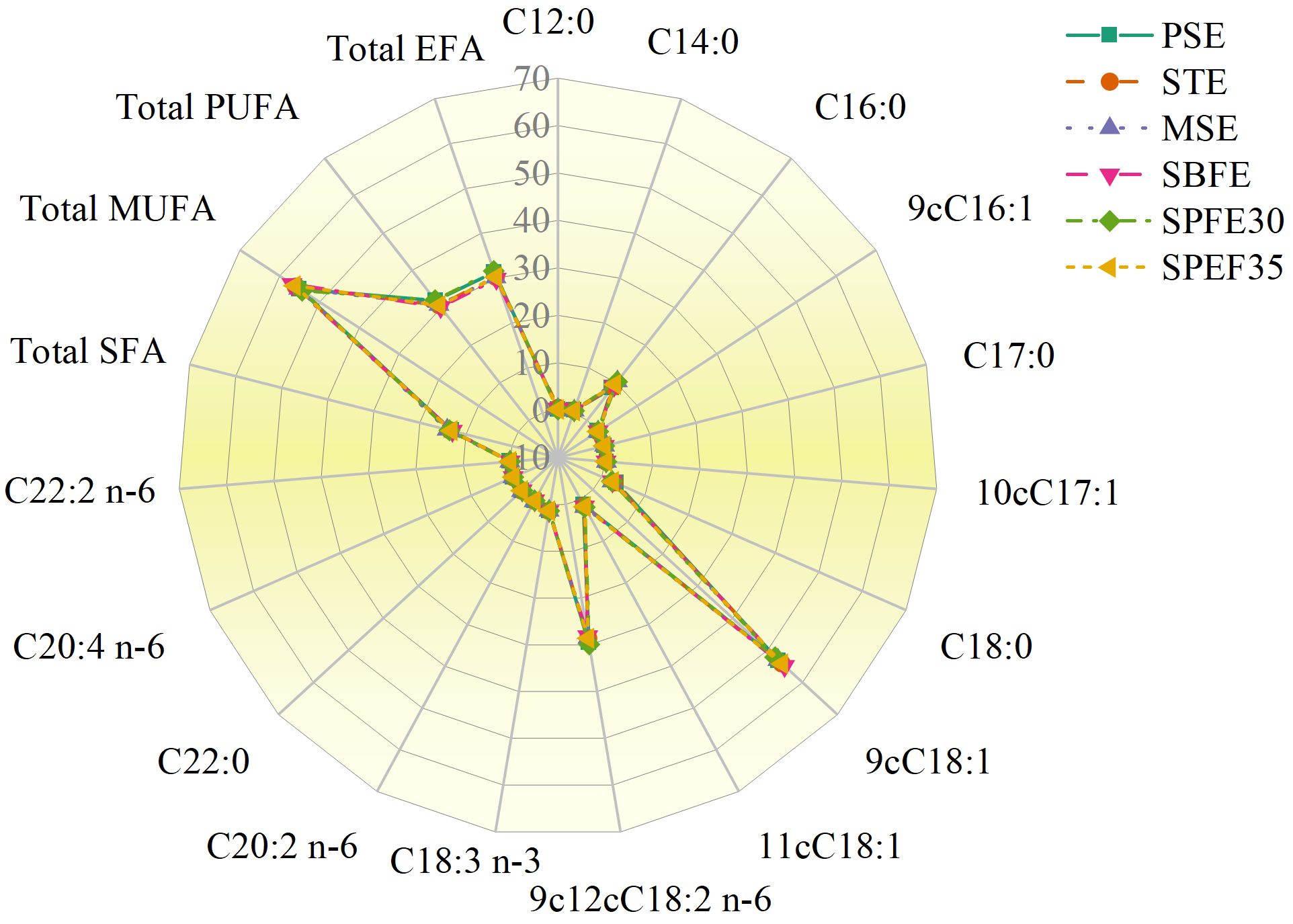
**

**Fig. S2.**

**

**

**Fig. S3.**

**

**

**Fig. S4.**

**
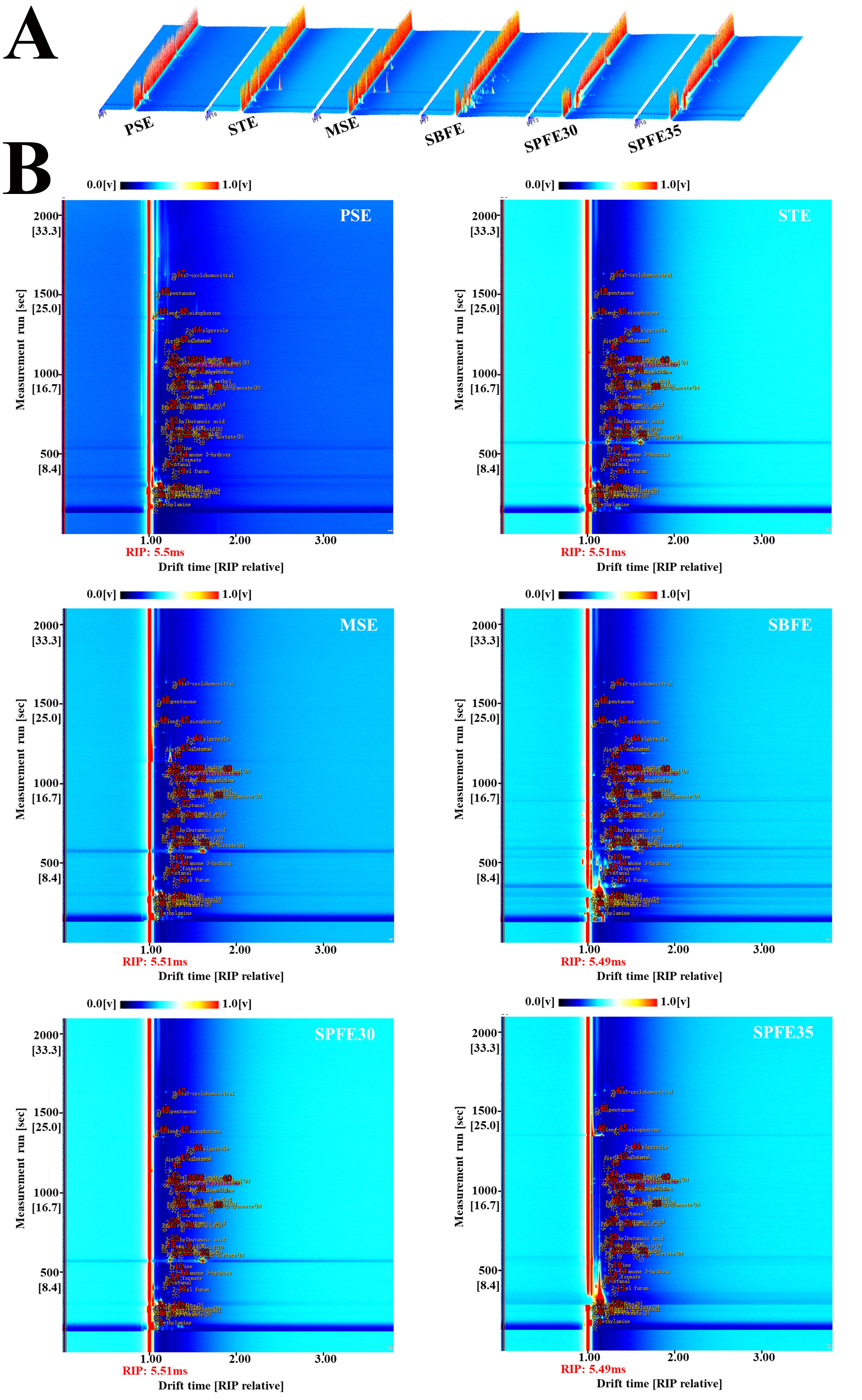
**

**Fig. S5.**

**
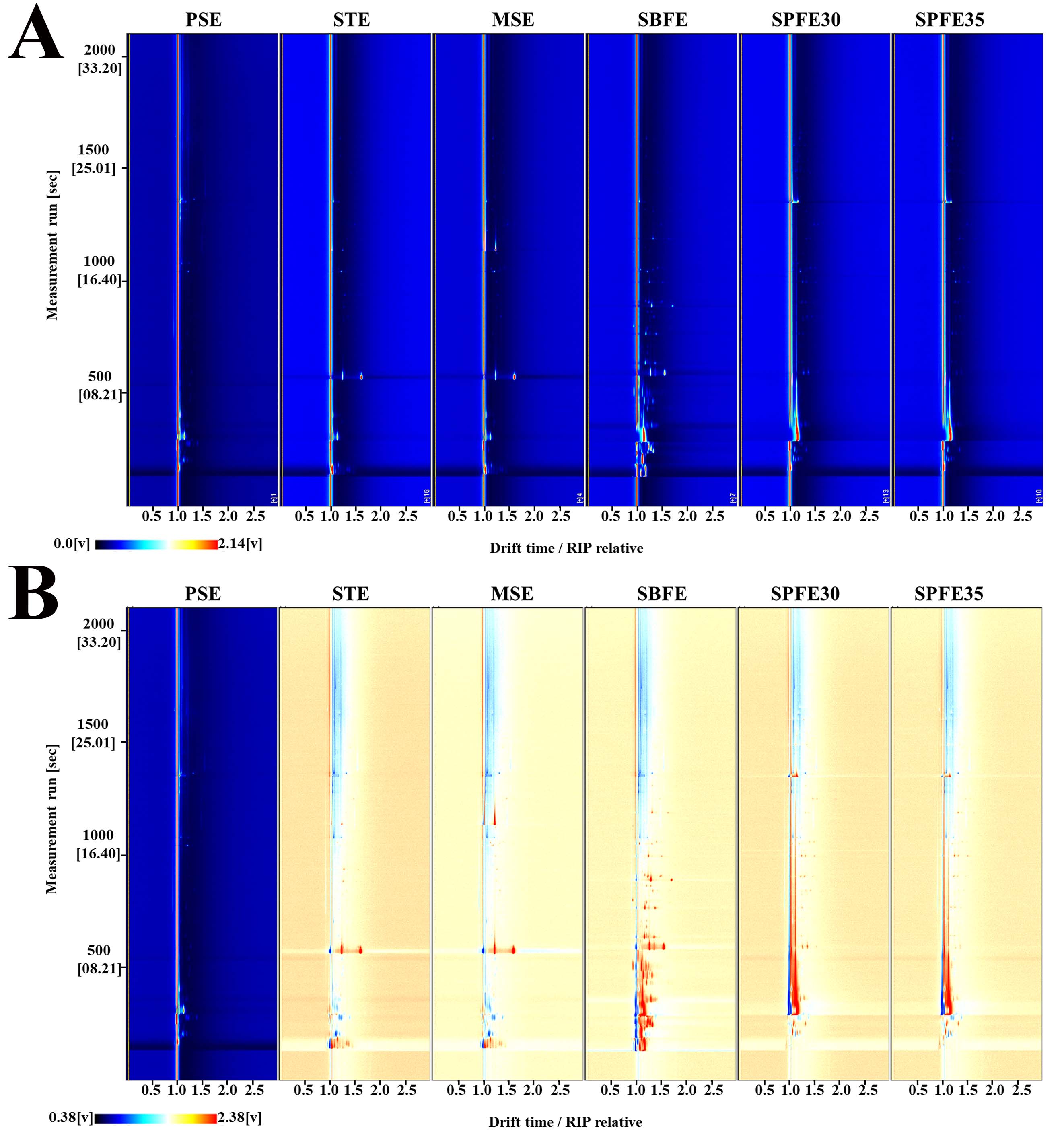
**

**Fig. S6.**

**Table Captions**

**Table S1. Differential lipid molecules in pecan oil extracted by different extraction methods**

| Lipids | VIP | P.value | RT (min) | m/z |
| --- | --- | --- | --- | --- |
| ACar(18:0) | 1.19391 | 4.132E-07 | 349.2285 | 428.3643466 |
| ACar(18:1) | 1.11081 | 1.906E-15 | 250.6895 | 426.3580411 |
| ACar(21:1) | 1.28345 | 2.7819E-08 | 513.0255 | 468.412485 |
| ACar(22:3) | 1.22177 | 0.0005591 | 629.603 | 478.3886528 |
| AcylGlcADG(12:0/12:0/22:0) | 1.14286 | 0.0058526 | 533.0515 | 953.7410044 |
| AcylGlcADG(20:4/22:6/22:6) | 1.29782 | 0.0086166 | 390.677 | 1173.761284 |
| Cer/ADS(d14:0/28:1) | 1.25452 | 2.9566E-07 | 496.701 | 710.6300491 |
| Cer/ADS(d14:0/29:1) | 1.25343 | 0.000017258 | 508.4 | 724.6479388 |
| Cer/ADS(d15:0/19:0) | 1.26303 | 0.000089774 | 383.5445 | 600.5222011 |
| Cer/ADS(d19:0/15:1) | 1.18931 | 0.0057299 | 366.5405 | 598.5054834 |
| Cer/ADS(d21:0/19:1) | 1.21419 | 5.7655E-07 | 474.983 | 682.5973608 |
| Cer/ADS(d21:0/20:0) | 1.15919 | 0.000012487 | 478.968 | 698.6298927 |
| Cer/ADS(d21:0/20:1) | 1.23633 | 1.4576E-06 | 486.109 | 696.6121178 |
| Cer/ADS(d23:0/19:0) | 1.20616 | 0.00030342 | 489.472 | 712.6467996 |
| Cer/ADS(d24:0/16:2) | 1.25914 | 0.000031961 | 462.469 | 680.5841647 |
| Cer/ADS(d24:0/19:0) | 1.2448 | 0.0039584 | 500.5515 | 726.6608594 |
| Cer/ADS(d25:0/19:1) | 1.23221 | 0.0000129 | 507.096 | 738.6540915 |
| Cer/ADS(d26:0/16:2) | 1.24549 | 2.6771E-07 | 484.858 | 708.6160141 |
| Cer/ADS(d27:0/15:1) | 1.23631 | 6.4715E-07 | 485.506 | 710.6213325 |
| Cer/AP(t14:0/16:1) | 1.27968 | 1.3565E-07 | 315.3555 | 512.4307616 |
| Cer/AP(t14:0/17:0) | 1.29248 | 6.6663E-08 | 338.336 | 528.4519745 |
| Cer/AP(t14:0/29:1) | 1.1904 | 7.4509E-06 | 496.034 | 740.6244569 |
| Cer/AP(t14:1/17:0) | 1.29171 | 1.1314E-08 | 338.329 | 526.4451854 |
| Cer/AP(t18:1/23:0) | 1.20493 | 0.00006747 | 495.971 | 712.6012297 |
| Cer/AS(d14:3/15:1) | 1.28003 | 7.8279E-15 | 294.261 | 476.3820336 |
| Cer/AS(d18:2/16:0) | 1.21029 | 0.0011405 | 363.2665 | 550.4838651 |
| Cer/AS(d24:2/18:0) | 1.25005 | 1.3011E-07 | 484.882 | 662.6081769 |
| Cer/EODS(d19:0/15:1/O/18:2) | 1.11706 | 0.0014576 | 509.694 | 860.7311721 |
| Cer/EOS(d15:1/24:2/O/18:1) | 1.10115 | 0.00018862 | 630.463 | 928.7961064 |
| Cer/EOS(d21:1/16:2/O/18:1) | 1.11805 | 0.0014541 | 628.677 | 900.7767179 |
| Cer/EOS(d21:1/16:2/O/20:2) | 1.11682 | 0.00021809 | 630.004 | 926.789846 |
| Cer/EOS(d23:1/14:0/O/18:2) | 1.13547 | 0.000033356 | 644.817 | 902.7927827 |
| Cer/EOS(d23:1/16:2/O/18:1) | 1.13254 | 0.000088064 | 645.3325 | 928.8046504 |
| Cer/EOS(d24:1/16:2/O/18:1) | 1.13167 | 0.007376 | 550.997 | 942.8087995 |
| Cer/NDS(d14:0/20:2) | 1.24555 | 0.000058304 | 385.4425 | 580.4940272 |
| Cer/NS(d18:2/16:0) | 1.32689 | 0.00099464 | 385.81 | 536.5031702 |
| DGTS(10:0/26:1) | 1.25228 | 1.6157E-06 | 473.323 | 766.651989 |
| DGTS(16:0/19:0) | 1.31036 | 6.3163E-06 | 477.898 | 754.6582503 |
| DGTS(17:0/17:0) | 1.25175 | 5.4578E-16 | 469.6125 | 740.6411475 |
| DGTS(17:0/18:1) | 1.20961 | 0.000023021 | 465.752 | 752.6398469 |
| DGTS(19:0/19:1) | 1.17219 | 0.0039352 | 494.374 | 794.6868787 |
| DGTS(19:2/19:2) | 1.14913 | 5.3198E-06 | 441.113 | 788.6440123 |
| DGTS(2:0/15:0) | 1.15563 | 1.1447E-07 | 75.5054 | 502.3744739 |
| DGTS(26:4/22:5) | 1.17977 | 0.00013849 | 610.225 | 918.7021313 |
| DGTS(6:0/27:0) | 1.35519 | 2.3473E-11 | 457.098 | 726.6228797 |
| DGTS(7:0/22:3) | 1.11566 | 0.00059583 | 323.14 | 664.5151124 |
| DGTS(8:0/22:6) | 1.1975 | 6.0052E-11 | 267.5725 | 672.5026312 |
| DGTS(9:0/22:5) | 1.12706 | 8.2148E-06 | 172.246 | 688.4976077 |
| FAHFA(18:1/18:2) | 1.10601 | 5.967E-07 | 405.7205 | 559.4721743 |
| FAHFA(18:1/20:3) | 1.13515 | 6.6049E-08 | 169.397 | 585.4868441 |
| FFA(17:1) | 1.12489 | 0.00023922 | 137.397 | 267.2326972 |
| FFA(21:0) | 1.11398 | 0.00033784 | 323.895 | 325.3107053 |
| GlcADG(14:0/22:3) | 1.2306 | 0.000047577 | 413.574 | 793.542699 |
| GlcADG(16:0/16:0) | 1.13079 | 0.0011302 | 426.0025 | 743.5495765 |
| GlcADG(19:2/19:2) | 1.10262 | 6.3913E-07 | 431.683 | 819.553087 |
| GlcADG(27:0/22:3) | 1.12597 | 0.000093554 | 468.3015 | 975.7508127 |
| GlcADG(27:0/22:5) | 1.24275 | 0.00025003 | 416.894 | 971.7192881 |
| HBMP(16:0/16:0/20:3) | 1.12426 | 0.0067646 | 491.448 | 1009.736639 |
| HBMP(16:1/16:1/16:1) | 1.10751 | 0.0017437 | 473.7755 | 953.7055283 |
| HexCer/AP(t14:0/19:1) | 1.16595 | 0.00015453 | 422.1225 | 716.5330211 |
| HexCer/AP(t14:0/35:1) | 1.19193 | 0.000008259 | 558.2115 | 986.8030847 |
| HexCer/AP(t14:1/23:1) | 1.16785 | 0.0019077 | 422.015 | 770.5412113 |
| HexCer/AP(t18:0/23:0) | 1.19891 | 5.9451E-06 | 449.566 | 876.6651298 |
| HexCer/NS(d25:3/14:0) | 1.17581 | 0.0012963 | 452.115 | 764.6133427 |
| LPE(18:1) | 1.10863 | 0.000018743 | 87.2727 | 480.3088559 |
| MGDG(16:0/18:1) | 1.25719 | 0.000299 | 390.016 | 801.5630965 |
| MGDG(16:0/18:2) | 1.24005 | 0.00043362 | 389.906 | 799.5573525 |
| MGDG(18:0/18:1) | 1.15231 | 0.00019089 | 439.081 | 829.6067687 |
| MGDG(18:0/18:2) | 1.14125 | 0.000090402 | 416.8515 | 827.5887982 |
| MGDG(18:1/18:1) | 1.11148 | 6.0564E-08 | 393.957 | 827.5796463 |
| MGDG(18:2/18:2) | 1.10134 | 0.00066018 | 343.684 | 823.5465257 |
| MGDG(18:3/18:3) | 1.10052 | 0.007361 | 316.618 | 819.5252126 |
| OxPE(18:1/20:4+4O(1Cyc)) | 1.10232 | 0.000040019 | 409.696 | 828.5187304 |
| OxPE(18:1/22:6+3O) | 1.13841 | 0.00095616 | 316.017 | 836.5185364 |
| PE(20:5e/14:0) | 1.11586 | 0.0012897 | 307.183 | 694.4977865 |
| PG(18:1/18:1) | 1.23473 | 0.00058827 | 355.036 | 792.5728264 |
| TAG(12:0/12:0/12:0) | 1.33022 | 3.4461E-10 | 489.6735 | 656.5813116 |
| TAG(12:0/12:0/14:0) | 1.25636 | 0.0018589 | 495.398 | 684.6102615 |
| TAG(12:0/12:0/18:1) | 1.34993 | 1.7924E-07 | 542.7 | 738.6624171 |
| TAG(12:0/12:0/19:3) | 1.1563 | 0.00024768 | 401.693 | 753.6138972 |
| TAG(12:0/12:0/21:2) | 1.10063 | 0.0021884 | 522.065 | 783.6502988 |
| TAG(12:0/12:1/16:0) | 1.36372 | 0.000041048 | 516.1945 | 710.6307667 |
| TAG(12:0/14:0/18:0) | 1.35404 | 1.7285E-07 | 607.3765 | 768.7099093 |
| TAG(12:0/20:2/20:2) | 1.14019 | 0.0017049 | 489.781 | 877.7258273 |
| TAG(12:1/12:1/16:5) | 1.20744 | 9.4326E-07 | 281.4225 | 703.4754069 |
| TAG(12:2/12:2/13:1) | 1.17369 | 3.2091E-11 | 196.505 | 665.4609411 |
| TAG(12:2/12:2/16:2) | 1.16736 | 1.5515E-07 | 490.193 | 705.5019326 |
| TAG(13:0/20:1/20:1) | 1.12268 | 0.005193 | 671.936 | 895.7731871 |
| TAG(13:1/13:1/18:1) | 1.14773 | 0.00037417 | 504.9875 | 767.6207898 |
| TAG(14:0/14:0/14:0) | 1.34196 | 6.0503E-10 | 569.773 | 740.6743818 |
| TAG(14:1/22:0/22:0) | 1.11599 | 0.001266 | 617.5685 | 967.8433758 |
| TAG(15:0/21:4/21:4) | 1.1385 | 0.0033306 | 532.1145 | 939.7608576 |
| TAG(17:2/18:2/18:3) | 1.17259 | 0.0056235 | 573.2685 | 880.717839 |
| TAG(18:4/19:1/19:1) | 1.11667 | 0.0057985 | 485.083 | 929.7659918 |

**Table S2.** **Composition of volatile compounds identified in pecan oil by GC-IMS**

| Compounds | Class | CAS | Formula | MW | RT | Rt (sec) | Da (a. u.) |
| --- | --- | --- | --- | --- | --- | --- | --- |
| (E)-2-Octenal | Aldehydes | 2548-87-0 | C8H14O | 126.2 | 1065.8 | 1189.508 | 1.32476 |
| 1-Heptanal | Aldehydes | 111-71-7 | C7H14O | 114.2 | 893.6 | 842.086 | 1.34244 |
| 1-Octanal (D) | Aldehydes | 124-13-0 | C8H16O | 128.2 | 997.5 | 1058.858 | 1.81742 |
| 1-Octanal (M) | Aldehydes | 124-13-0 | C8H16O | 128.2 | 997.8 | 1059.539 | 1.41205 |
| 2,3-Butanediol | Alcohols | 513-85-9 | C4H10O2 | 90.1 | 783.9 | 592.147 | 1.36705 |
| 2-Acetylpyrrole | Others | 1072-83-9 | C6H7NO | 109.1 | 1095.6 | 1251.512 | 1.47976 |
| 2-Butanone (D) | Ketones | 78-93-3 | C4H8O | 72.1 | 591 | 265.137 | 1.24061 |
| 2-Butanone (M) | Ketones | 78-93-3 | C4H8O | 72.1 | 591.4 | 265.445 | 1.06289 |
| 2-Butanone 3-hydroxy | Ketones | 513-86-0 | C4H8O2 | 88.1 | 737.7 | 466.24 | 1.3247 |
| 2-Ethyl furan | Furan | 3208-16-0 | C6H8O | 96.1 | 687.8 | 360.791 | 1.30129 |
| 2-Ethyl-6-methylpyrazine (D) | Pyrazines | 13925-03-6 | C7H10N2 | 122.2 | 990.4 | 1043.078 | 1.21274 |
| 2-Ethyl-6-methylpyrazine (M) | Pyrazines | 13925-03-6 | C7H10N2 | 122.2 | 990.6 | 1043.508 | 1.18377 |
| 2-Methyl-2-propenal | Aldehydes | 78-85-3 | C4H6O | 70.1 | 558 | 238.635 | 1.22195 |
| 2-Pentyl furan | Furan | 3777-69-3 | C9H14O | 138.2 | 969.8 | 996.541 | 1.25023 |
| 2-Propanone | Ketones | 67-64-1 | C3H6O | 58.1 | 517 | 209.442 | 1.11121 |
| 3-Ethylpyridine | Pyrazines | 536-78-7 | C7H9N | 107.2 | 969.9 | 996.859 | 1.51611 |
| 3-Heptanone, 5 methyl | Ketones | 541-85-5 | C8H16O | 128.2 | 942 | 937.209 | 1.28446 |
| 3-Methylbutanoic acid (D) | Acids | 503-74-2 | C5H10O2 | 102.1 | 873.2 | 789.747 | 1.21644 |
| 3-Methylbutanoic acid (M) | Acids | 503-74-2 | C5H10O2 | 102.1 | 827.2 | 681.181 | 1.21347 |
| 4-Ketoisophorone | Ketones | 1125-21-9 | C9H12O2 | 152.2 | 1146.6 | 1365.053 | 1.32525 |
| alpha-Phellandrene | Alkenes | 99-83-2 | C10H16 | 136.2 | 1008.5 | 1078.982 | 1.224 |
| Benzenemethanol | Alcohols | 100-51-6 | C7H8O | 108.1 | 998.2 | 1060.133 | 1.50021 |
| beta-Homocyclocitral | Aldehydes | 472-66-2 | C11H18O | 166.3 | 1241.3 | 1603.895 | 1.29602 |
| Butanal | Aldehydes | 123-72-8 | C4H8O | 72.1 | 580.2 | 256.153 | 1.2864 |
| Butanoic acid (D) | Acids | 107-92-6 | C4H8O2 | 88.1 | 805.9 | 636.061 | 1.3636 |
| Butanoic acid (M) | Acids | 107-92-6 | C4H8O2 | 88.1 | 806.2 | 636.598 | 1.17187 |
| Butyl acetate (D) | Esters | 123-86-4 | C6H12O2 | 116.2 | 777.4 | 572.779 | 1.61969 |
| Butyl acetate (M) | Esters | 123-86-4 | C6H12O2 | 116.2 | 779.7 | 579.525 | 1.23692 |
| Butyl formate | Esters | 592-84-7 | C5H10O2 | 102.1 | 724.5 | 435.445 | 1.21428 |
| Butyl propanoate (D) | Esters | 590-01-2 | C7H14O2 | 130.2 | 918.4 | 889.525 | 1.71243 |
| Butyl propanoate (M) | Esters | 590-01-2 | C7H14O2 | 130.2 | 919.9 | 892.437 | 1.29645 |
| Butyric acid (D) | Acids | 107-92-6 | C4H8O2 | 88.1 | 863.4 | 765.133 | 1.38444 |
| Butyric acid (M) | Acids | 107-92-6 | C4H8O2 | 88.1 | 864.1 | 766.946 | 1.18232 |
| Cyclopentanone | Ketones | 120-92-3 | C5H8O | 84.1 | 1198.2 | 1490.177 | 1.10868 |
| Diethyl malonate | Esters | 105-53-3 | C7H12O4 | 160.2 | 1043.4 | 1144.915 | 1.2376 |
| Dimethyl trisulfide | Sulfur compounds | 3658-80-8 | C2H6S3 | 126.3 | 970.4 | 997.76 | 1.32525 |
| Dimethylamine | Others | 124-40-3 | C2H7N | 45.1 | 405.9 | 147.049 | 1.08545 |
| Ethyl acetate (D) | Esters | 141-78-6 | C4H8O2 | 88.1 | 566.4 | 245.108 | 1.33402 |
| Ethyl acetate (M) | Esters | 141-78-6 | C4H8O2 | 88.1 | 565.9 | 244.778 | 1.09423 |
| Ethyl formate (D) | Esters | 109-94-4 | C3H6O2 | 74.1 | 521.9 | 212.744 | 1.21792 |
| Ethyl formate (M) | Esters | 109-94-4 | C3H6O2 | 74.1 | 522.4 | 213.074 | 1.0728 |
| Hexanal (D) | Aldehydes | 66-25-1 | C6H12O | 100.2 | 784.1 | 593.011 | 1.55327 |
| Hexanal (M) | Aldehydes | 66-25-1 | C6H12O | 100.2 | 785.7 | 596.036 | 1.26803 |
| n-Pentanal | Aldehydes | 110-62-3 | C5H10O | 86.1 | 710.1 | 404.324 | 1.17594 |
| Pentanoic acid (D) | Acids | 109-52-4 | C5H10O2 | 102.1 | 927.8 | 908.089 | 1.50016 |
| Pentanoic acid (M) | Acids | 109-52-4 | C5H10O2 | 102.1 | 927 | 906.633 | 1.24167 |
| p-Xylene | Others | 106-42-3 | C8H10 | 106.2 | 1147 | 1365.843 | 1.08199 |
| Pyridine | Others | 110-86-1 | C5H5N | 79.1 | 752.8 | 504.127 | 1.25914 |
